# Single-cell analysis and EpCAM-based microfluidic chip detection of PD-L1 on circulating tumor cells for prognostic prediction in epithelial ovarian cancer

**DOI:** 10.3389/fonc.2026.1824350

**Published:** 2026-08-10

**Authors:** Lin Zhang, Hongqing Lyu, Xia Tong, Shuai Lou

**Affiliations:** 1 Department of Gynecology, Jinyi Group Jinhua Maternal and Child Health Hospital, Jinhua, China; 2 Department of Gynecology, Affiliated Jinhua Hospital, Zhejiang University School of Medicine, Jinhua, China

**Keywords:** circulating tumor cells, CTCs, epithelial ovarian cancer, HB-Chip, PD-L1, prognosis, single-cell RNA sequencing

## Abstract

**Background:**

The prognosis of epithelial ovarian cancer (EOC) after comprehensive treatment remains unsatisfactory. This study aimed to evaluate the feasibility of enriching and detecting circulating tumor cells (CTCs) in peripheral blood from EOC patients using EpCAM antibody-based microfluidic chips, investigate the prognostic value of CTC PD-L1 expression, and analyze the cellular heterogeneity of ovarian cancer through re-analysis of publicly available single-cell RNA sequencing data.

**Methods:**

Seventy-two EOC patients undergoing surgery at Jinhua Maternal and Child Health Hospital (2020.07–2023.12) were enrolled. CTCs were enriched and PD-L1 detected via CytoSorter with EpCAM-coated HB-Chip. Clinicopathological associations were tested by chi-square/Fisher’s exact tests; paired CTC counts compared by Wilcoxon signed-rank test. Prognostic analyses used Kaplan-Meier curves (log-rank test) and Cox proportional hazards regression. Single-cell RNA-seq data (GEO: GSM8072347) were re-analyzed for tumor microenvironment PD-L1 expression patterns. qPCR and flow cytometry validated PD-L1 expression in three EOC cell lines.

**Results:**

Pre- and postoperative PD-L1+ CTC detection rates were 56.9% (41/72) and 45.8% (33/72), with significant postoperative decline (P < 0.0001). Pre-operative PD-L1+ CTC <2/4 mL predicted shorter PFS (median: 1.5 vs 3.3 months; P = 0.008), and remained independent in multivariate Cox regression (adjusted HR = 2.35, 95% CI: 1.39–3.96, P = 0.001), while FIGO stage III/IV was the strongest predictor (adjusted HR = 2.66, 95% CI: 1.55–4.57, P < 0.001). Total CTC count showed no PFS association (P = 0.858). scRNA-seq re-analysis identified distinct cell populations and CD274 expression patterns across the tumor microenvironment. IFN-γ-induced PD-L1 upregulation was confirmed by qPCR and surface protein validated by flow cytometry across EOC cell lines.

**Conclusion:**

The EpCAM antibody-based HB-Chip can achieve enrichment and detection of CTCs in peripheral blood from EOC patients. Pre-operative PD-L1+ CTC count is an independent prognostic factor for progression-free survival. CTC PD-L1 detection may assist in postoperative prognostic evaluation. Re-analysis of publicly available single-cell transcriptomic data further elucidated tumor microenvironment heterogeneity, and *in vitro* experiments provided supportive evidence for immune-related gene regulation in ovarian cancer cells.

## 1 Introduction

Epithelial ovarian cancer (EOC) is a common gynecological malignancy, accounting for approximately 90% of all types of ovarian malignancies (1). In recent years, the incidence of EOC has shown an increasing trend year by year, and it remains the disease with the highest mortality rate in gynecology. Although the clinical application of cytoreductive surgery has greatly improved patient prognosis, the overall recurrence and mortality rates of EOC remain high, and the prognostic results after comprehensive treatment are still unsatisfactory. The five-year survival rate for advanced-stage EOC patients remains dismally low at approximately 30-40% (1, 2), highlighting the urgent need for novel diagnostic and prognostic biomarkers (3). The heterogeneous nature of EOC, comprising multiple histological subtypes with distinct molecular profiles and clinical behaviors, further complicates treatment strategies and prognostic assessment. Therefore, finding predictive factors for early evaluation of EOC patient prognosis has important clinical significance.

Clinical studies have shown that the expression of programmed cell death-ligand 1 (PD-L1) on circulating tumor cells (CTCs) (4) is a potential independent prognostic predictive biomarker that may assist in prognostic evaluation of lung cancer (3, 4), liver cancer (5, 6), breast cancer (7), and other solid tumors (8), cervical cancer (9, 10), and other tumors. CTCs represent a unique liquid biopsy approach that enables real-time monitoring of tumor evolution (11) and treatment response through minimally invasive blood sampling. The detection of PD-L1 expression on CTCs is particularly significant as it not only provides prognostic information but also potentially guides immunotherapy decisions, especially considering the emerging role of immune checkpoint inhibitors in ovarian cancer treatment. To the best of our knowledge, published studies on CTC PD-L1 detection and its clinical application specifically in ovarian cancer remain scarce. While CTC PD-L1 has been investigated as a prognostic marker in other solid tumors including lung cancer (3, 4), liver cancer (5, 6), and breast cancer (7), its prognostic and clinical role in EOC remains insufficiently characterized, representing an important knowledge gap warranting dedicated investigation.

The advent of single-cell RNA sequencing (scRNA-seq) technology has revolutionized our understanding of tumor heterogeneity and the tumor microenvironment (TME) at high resolution. Critically, the single-cell resolution afforded by scRNA-seq enables dissection of PD-L1 expression heterogeneity across individual cell populations—including epithelial, immune, and stromal cell populations that may provide relevant biological context—at a granularity not achievable by bulk transcriptomic approaches, thereby complementing interpretation of the prognostic significance of PD-L1+ CTCs detected in peripheral blood. This powerful technology enables the dissection of cellular diversity within tumors, revealing distinct cell populations, their transcriptional states, and dynamic interactions that cannot be captured by bulk RNA sequencing approaches (12). In the context of ovarian cancer, scRNA-seq has begun to unveil the complex ecosystem comprising not only heterogeneous tumor cells but also cancer-associated fibroblasts (CAFs), tumor-infiltrating lymphocytes, myeloid cells, and endothelial cells, each playing crucial roles in tumor progression, metastasis, and therapeutic resistance (13). Recent single-cell studies have identified novel ovarian cancer cell states associated with chemoresistance, stem-like properties, and epithelial-mesenchymal transition (EMT), providing insights into the molecular mechanisms underlying treatment failure and disease recurrence (14).

The integration of CTC analysis with single-cell transcriptomic profiling represents a complementary approach to characterizing PD-L1 expression heterogeneity in the ovarian cancer microenvironment and providing biological context for the observed CTC findings. Future paired single-cell analyses of primary tumors and CTCs may help identify transcriptional programs associated with tumor cell dissemination and survival in circulation, potentially informing therapeutic strategies against metastatic spread, which is the primary cause of mortality in EOC patients.

The main purpose of this study is to explore the feasibility of enriching and detecting CTCs in peripheral blood of EOC patients using epithelial cell adhesion molecule (EpCAM) antibody-modified herringbone structure microfluidic chip (HB-Chip), and to explore the application value of CTC PD-L1 expression detection before and after surgery in prognostic prediction of EOC patients. This approach addresses the challenge of epithelial-mesenchymal plasticity in CTCs, as tumor cells undergoing EMT may lose epithelial markers while gaining mesenchymal characteristics, making them undetectable by EpCAM-based capture alone.

Additionally, to deeply understand the cellular composition and heterogeneity of epithelial ovarian cancer at the single-cell level and provide a molecular basis for the origin and heterogeneity of CTCs, this study re-analyzed a publicly available single-cell RNA sequencing dataset from ovarian cancer tissue samples (GEO: GSM8072347) to investigate the expression patterns of CD274/PD-L1 and related immune checkpoint genes across distinct cell types in the ovarian cancer microenvironment, thereby providing biological context for the prognostic observations in the clinical cohort. Additionally, quantitative real-time PCR (qPCR) was performed in EOC cell lines following IFN-γ stimulation to assess PD-L1 and related gene expression changes at the transcriptional level.

## 2 Materials and methods

### 2.1 Study subjects

Seventy-two patients with epithelial ovarian cancer who underwent surgical treatment at Jinhua Maternal and Child Health Hospital from July 2020 to December 2023 were enrolled, with a mean age of 60.3 ± 10.3 years. Histological subtypes included 42 serous carcinoma, 16 mucinous carcinoma, 10 endometrioid adenocarcinoma, and 4 clear cell carcinoma. According to the International Federation of Gynecology and Obstetrics (FIGO) staging criteria (2014), there were 18 stage I, 16 stage II, 23 stage III, and 15 stage IV patients. Twenty-seven patients had poorly differentiated tumors and 45 had moderately or well differentiated tumors. Lymph node metastasis was present in 33 patients and absent in 39 patients. Residual disease after surgery was categorized as R0 in 40 patients, R1 in 22 patients, and R2 in 10 patients. The most common chemotherapy regimen was carboplatin plus paclitaxel (60%), followed by cisplatin plus paclitaxel (30%). All patients completed at least 3 cycles of adjuvant platinum-based chemotherapy. Additionally, relevant comorbidities included hypertension in 18 patients (25.0%), diabetes mellitus in 9 patients (12.5%), and other chronic conditions in 14 patients (19.4%). Body mass index (BMI) data were available for 68 patients (mean 23.8 ± 3.5 kg/m²). All patients had an Eastern Cooperative Oncology Group (ECOG) performance status of 0–1 at enrollment. Pre-operative CA125 levels ranged from 8.5 to 3,420 U/mL (median 128.5 U/mL).

Inclusion criteria: ① No history of other malignant tumors; ② No preoperative radiotherapy or chemotherapy; ③ Complete clinical data; ④ Voluntary signing of informed consent and willingness to accept CTC PD-L1 detection and follow-up.

Exclusion criteria: ① Incomplete clinical data; ② Severe cardiac, hepatic, and renal insufficiency; ③ History of other malignant tumors; ④ Unwillingness to sign informed consent.

This study was approved by the Research Ethics Committee of our hospital (Ethics Approval No.: 2022KYJZ026).

### 2.2 CTC detection method

Peripheral blood (4 mL) was collected from the cubital vein of all enrolled patients before and after surgery. The herringbone structure microfluidic chip (HB-Chip) was coated with anti-EpCAM capture antibody (BioLegend, San Diego, CA, USA). The HB-Chip utilizes herringbone groove microstructures to generate chaotic mixing within microfluidic channels, increasing the probability of CTC-antibody interactions. Published validation studies using this platform have reported capture efficiencies of 85-95% for EpCAM-expressing cancer cells (15, 16). CTC enrichment and PD-L1 detection were performed using the CytoSorter Circulating Tumor Cell Separator and CTC PD-L1 Detection Kit (Hangzhou Watson Biotechnology Co., Ltd., Hangzhou, China) according to the manufacturer**’**s instructions. Immunofluorescence staining was performed as follows: (1) cell permeabilization at room temperature in the dark for 10 minutes; (2) PanCK-fluorescein isothiocyanate (FITC), CD45-phycoerythrin (PE), and PD-L1-cyanine 5 (Cy5) fluorescent antibodies (all provided in the CTC PD-L1 Detection Kit, Hangzhou Watson Biotechnology Co., Ltd., Hangzhou, China) incubation at room temperature in the dark for 1 hour; (3) Hoechst 33342 (Sigma-Aldrich, St. Louis, MO, USA) nuclear staining. PanCK was labeled with FITC (green channel) for CTC identification. A Nikon Ti-E inverted fluorescence microscope(Nikon, Tokyo, Japan) was used for identification of CTC and PD-L1 immunofluorescence staining results. The EpCAM-based enrichment strategy was selected because EpCAM is widely expressed in epithelial ovarian cancers (>90% of cases), providing broad applicability for CTC capture. The herringbone groove design generates passive chaotic mixing that enhances CTC-surface contact frequency compared to linear microfluidic channels, thereby improving capture efficiency without requiring external pumps or complex instrumentation.

CTC identification criteria: PD-L1+ CTC: PD-L1-Cy5+, PanCK-FITC+, Hoechst 33342+, CD45-PE-; PD-L1- CTC: PD-L1-Cy5-, PanCK-FITC+, Hoechst 33342+, CD45-PE-; White blood cell (WBC): PD-L1-Cy5-, PanCK-FITC-, Hoechst 33342+, CD45-PE+. CTC counts were reported as number per 4 mL of peripheral blood. Cutoff values (<2/4 mL for preoperative and **<**1/4 mL for postoperative PD-L1+ CTC) were determined based on clinically relevant thresholds approximating the median values of PD-L1+ CTC counts. At least two independent observers reviewed all immunofluorescence images, and discrepancies were resolved by consensus.

### 2.3 Follow-up method

Patient follow-up data were mainly collected through telephone, WeChat, and hospital re-examination. The follow-up deadline was August 2024, with a mean follow-up duration of 27.6 ± 12.2 months (range: 3.0-54.0 months). Disease progression status, survival status, and treatment information after surgery were recorded. Follow-up assessments were conducted at 3-month intervals for the first 2 years and every 6 months thereafter. Disease progression was determined according to the Response Evaluation Criteria in Solid Tumors version 1.1 (RECIST v1.1) based on imaging studies (CT or MRI), supplemented by serum CA125 levels according to Gynecologic Cancer InterGroup (GCIG) criteria and clinical symptom assessment. Progression events were independently adjudicated by two gynecologic oncologists blinded to CTC results.

Progression-free survival (PFS) was defined as the time interval from when the patient began receiving surgical treatment to disease progression (such as continued tumor growth, spread, or appearance of new lesions) or death from any cause. Radiographic progression was defined as ≥20% increase in the sum of target lesion diameters (RECIST v1.1), and CA125 progression was defined according to GCIG criteria (doubling of CA125 from the upper limit of normal or nadir, confirmed by a second sample).

### 2.4 Single-cell RNA sequencing data acquisition and preprocessing

To complement the CTC analysis and provide single-cell-level molecular context for PD-L1 expression in the ovarian cancer microenvironment, a publicly available single-cell RNA sequencing (scRNA-seq) dataset was re-analyzed (Gene Expression Omnibus accession number GSM8072347). These data were generated from primary ovarian cancer tissue specimens (not from the patients in the clinical CTC cohort) and were deposited in GEO by the original data generators. First, strict quality control was performed on the raw data, including filtering low-quality cells and doublets to ensure data reliability. Subsequently, by identifying highly variable genes and genes with large contributions to principal components, the main sources of variation in the data were captured. Principal component analysis (PCA) was used for preliminary revelation of cell population structure. After determining the optimal number of principal components through JackStraw analysis and elbow plots, nonlinear dimensionality reduction methods such as UMAP and t-SNE were used for cell dimensionality reduction and clustering, clearly dividing cells into multiple subpopulations. Finally, based on specific gene expression, precise cell type annotation was performed on these cell subpopulations.

To characterize PD-L1 (CD274) expression at single-cell resolution, we analyzed the expression profiles of seven immune checkpoint genes (CD274/PD-L1, PDCD1, PDCD1LG2, CTLA4, LAG3, TIGIT, HAVCR2) across the annotated cell types using dot plot visualization (showing mean expression and fraction of expressing cells) and hierarchical clustering heatmap analysis.

### 2.5 Single-cell data analysis: gene co-expression network and pseudotime analysis

Based on cell type annotation, this study further conducted gene co-expression network analysis and pseudotime analysis. A weighted gene co-expression network was constructed, and optimal parameters for network construction were determined through soft threshold selection. hdWGCNA analysis identified multiple gene modules and analyzed correlations between modules, with some modules closely related to cell proliferation processes. Differential expression analysis was performed on genes in proliferation modules, and volcano plots of specific modules were displayed by different cell types. Additionally, this study particularly focused on telomere maintenance genes, analyzing their expression overview across different cell types, interaction networks with cell types, and visualizing telomere activity and functional classification of related genes. To reveal the dynamic evolution process of ovarian cancer cells during disease progression, pseudotime analysis was performed. By inferring cell pseudotime trajectories, the continuity of ovarian cancer cell transition from one state to another was revealed, and the trajectories and density distributions of different cell types along the pseudotime axis were depicted. Meanwhile, expression changes of specific genes (such as HES4, INTS11, ISG15, NOC2L, SDF4, UBE2J2) under different pseudotime states were analyzed to characterize gene expression dynamics along the inferred pseudotime trajectory.

### 2.6 Cell culture and quantitative real-time PCR

Three human EOC cell lines were used: SKOV3 (ATCC HTB-77, Manassas, VA, USA; high-grade serous), A2780 (ECACC 93112519, Porton Down, Salisbury, UK; endometrioid), and OVCAR3 (ATCC HTB-161, Manassas, VA, USA; high-grade serous). All cell lines were authenticated by short tandem repeat profiling and maintained in RPMI-1640 medium (Gibco, Grand Island, NY, USA) supplemented with 10% fetal bovine serum (Gibco, Grand Island, NY, USA) and 1% penicillin–streptomycin (Gibco, Grand Island, NY, USA) at 37 °C in a humidified 5% CO_2_ atmosphere. To investigate whether PD-L1 expression is dynamically regulated by immune signals across EOC subtypes, a property relevant to understanding PD-L1 heterogeneity on CTCs, cells were exposed to interferon-γ (IFN-γ, 10 ng/mL; PeproTech, Cranbury, NJ, USA) for 24 h, while untreated cells served as controls. Total RNA was isolated using TRIzol reagent (Invitrogen, Carlsbad, CA, USA), and 1 μg RNA was reverse-transcribed into cDNA with a PrimeScript RT kit (Takara Bio, Kusatsu, Japan). Quantitative PCR was performed using SYBR Green Master Mix (Applied Biosystems, Foster City, CA, USA) on an ABI 7500 system(Applied Biosystems, Foster City, CA, USA), and GAPDH was used as the internal reference. Relative expression levels were calculated using the 2^−ΔΔCt^ method. Primer sequences were: PD-L1 F 5′-TGGCATTTGCTGAACGCATTT-3′, R 5′-TGCAGCCAGGTCTAATTGTTTT-3′; HLA-DRA F 5′-TGTGGTGGTTGAGACTGCTG-3′, R 5′-TGCAGGAGTCTTGGGTGTAG-3′; CXCL10 F 5′-GAAAGCAGTTAGCAAGGAAAGGT-3′, R 5′-GACATATACTCCATGTAGGGGAAG-3′; IRF1 F 5′-GGCTGCTATTTCCTCCTCCA-3′, R 5′-CCTGCTCTGGTCTTTCACCT-3′; MKI67 F 5′-AAGCCCTCCAGCTCCTGTT-3′, R 5′-ATGAGGCTGAGGAGGAGTTG-3′; and GAPDH F 5′-GGAGCGAGATCCCTCCAAAAT-3′, R 5′-GGCTGTTGTCATACTTCTCATGG-3′. Each condition was tested in triplicate, and all reactions showed single, specific melt peaks.

For flow cytometry analysis of surface PD-L1 protein, cells were harvested, washed with PBS (Gibco, Grand Island, NY, USA), and incubated with PE-conjugated anti-human PD-L1 antibody (clone 29E.2A3, BioLegend, San Diego, CA, USA) at 4 °C for 30 min in the dark. After washing, cells were resuspended in 300 μL of flow cytometry staining buffer (eBioscience, Thermo Fisher Scientific, San Diego, CA, USA) and analyzed on a BD FACSCanto II flow cytometer (BD Biosciences, Franklin Lakes, NJ, USA). Data were processed using FlowJo™ v10.8 software (BD Life Sciences, Ashland, OR, USA). Median fluorescence intensity (MFI) was used to quantify surface PD-L1 protein levels.

### 2.7 Statistical methods

Statistical analyses were performed using GraphPad Prism 9.0 (GraphPad Software, San Diego, CA, USA), SPSS 26.0 (IBM Corp., Armonk, NY, USA), and R version 4.2 (survival and survminer packages). Continuous variables were summarized as mean ± standard deviation or median (range), as appropriate, after assessment of normality using the Shapiro–Wilk test and Q–Q plots. Categorical variables were summarized as frequencies and percentages and compared using the chi-square test or Fisher’s exact test when any expected cell count was <5. Paired preoperative and postoperative CTC counts were compared using the Wilcoxon signed-rank test. Progression-free survival was estimated using the Kaplan–Meier method and compared using the log-rank test. Cox proportional hazards regression was used for univariable and multivariable analyses. Variables with P < 0.10 in univariable analysis, together with clinically relevant covariates (age, FIGO stage, lymph node metastasis, and CA125 level), were considered for the multivariable model. Hazard ratios (HR) and 95% confidence intervals (CI) were reported. The proportional hazards assumption was assessed using Schoenfeld residuals. All tests were two-sided, and P < 0.05 was considered statistically significant.

## 3 Results

### 3.1 Preoperative and postoperative CTC PD-L1 expression detection results

**Figure 1:**
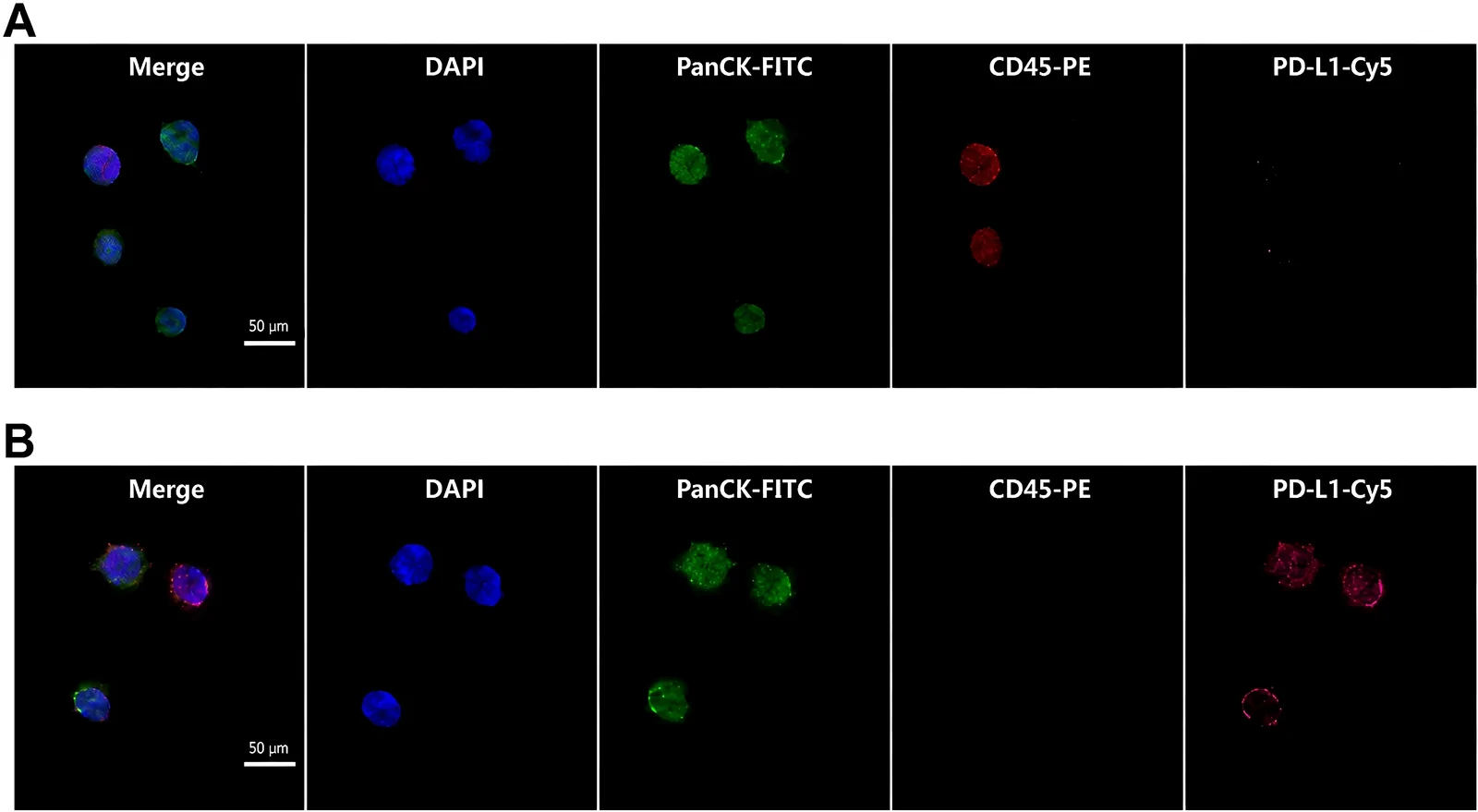
Immunofluorescence identification of CTC PD-L1 in peripheral blood of patients with epithelial ovarian cancer. **(A)** PD-L1-negative CTC: Hoechst 33342 (DAPI), PanCK-FITC (CTC marker), PD-L1-Cy5, CD45-PE (WBC exclusion), Merge, Brightfield. **(B)** PD-L1-positive CTC: same channels showing PD-L1-Cy5 positive signal. PD-L1+ CTC = Cy5+/FITC+/Hoechst+/PE-. Scale bar: 50 μm.

**Figure 2:**
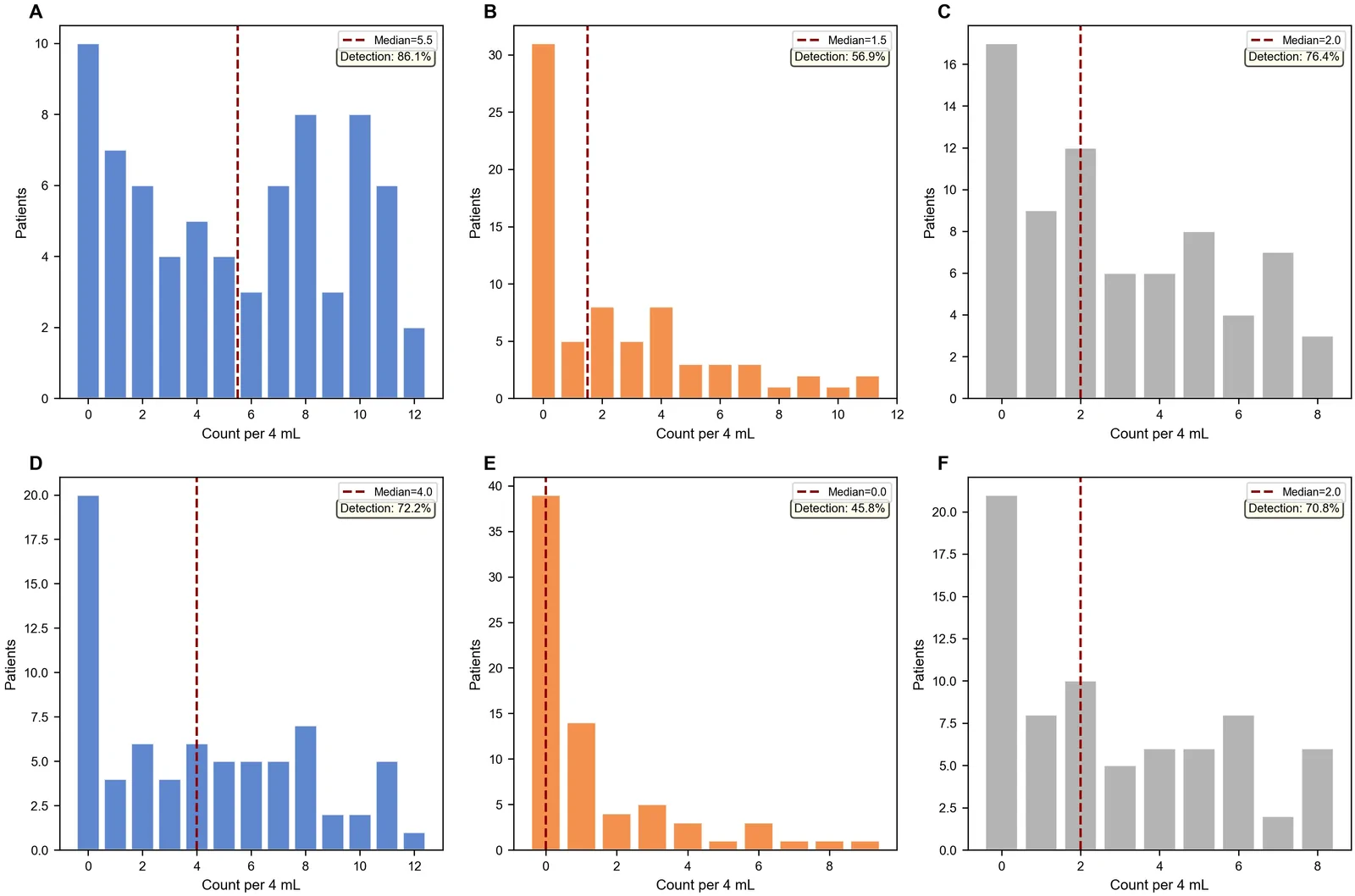
Quantitative distribution of CTCs and PD-L1+ CTCs test results in patients with epithelial ovarian cancer before and after operation (n=72). **(A)** Pre-op total CTC count (detection 86.1%, 62/72, median = 6/4 mL). **(B)** Pre-op PD-L1-positive CTC count (detection 56.9%, 41/72, median = 2/4 mL). **(C)** Pre-op PD-L1- CTC. **(D)** Post-op total CTC count (detection 72.2%, 52/72, median = 4/4 mL). **(E)** Post-op PD-L1-positive CTC count (detection 45.8%, 33/72, median = 0/4 mL). **(F)** Post-op PD-L1- CTC.

**Figure 3:**
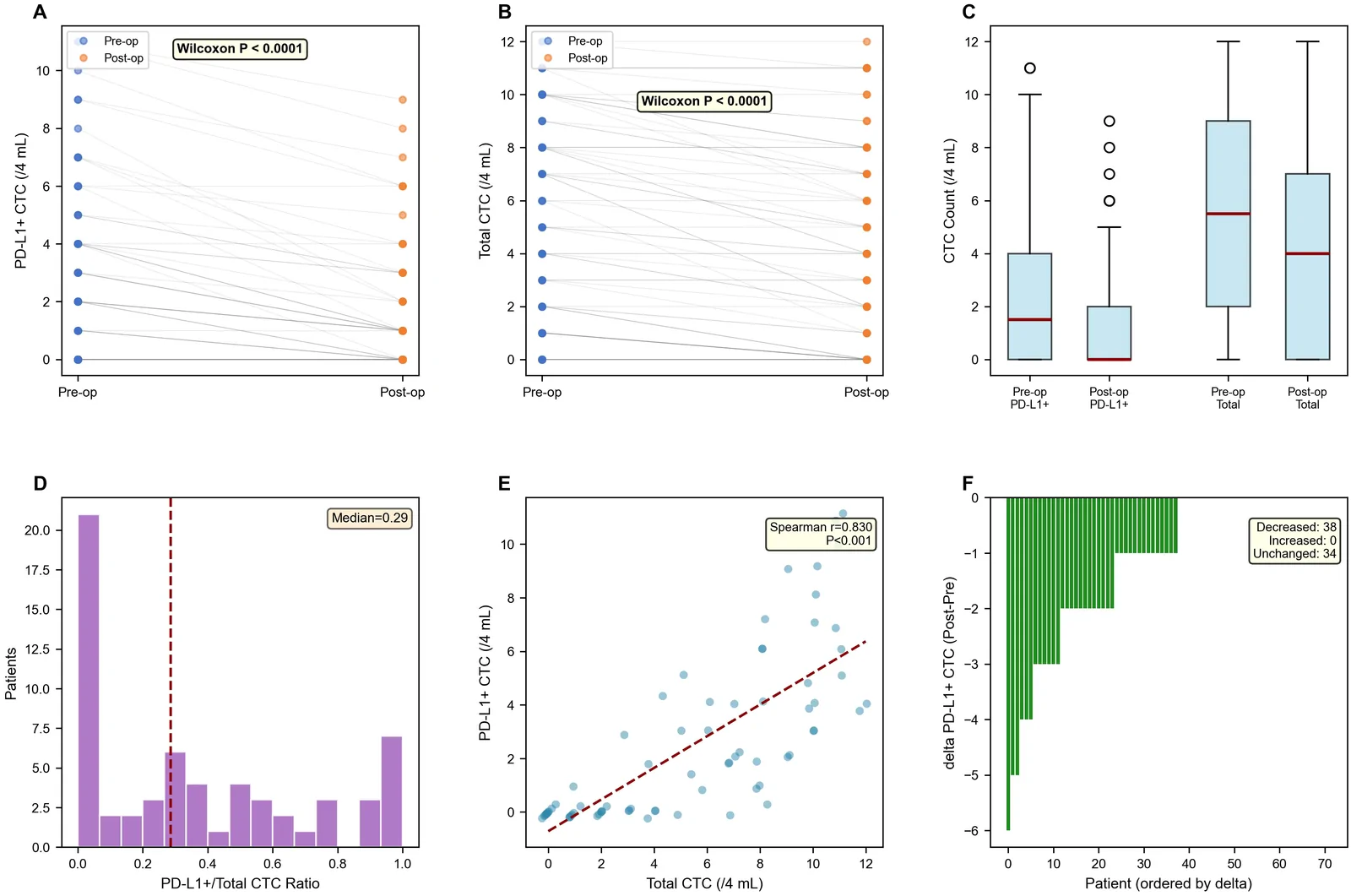
Quantitative changes in CTCs and PD-L1+ CTCs test results in patients with epithelial ovarian cancer before and after operation (n=72). **(A)** PD-L1-positive CTC count paired line plot (Pre vs Post), Wilcoxon signed-rank test, P < 0.0001. **(B)** Total CTC count paired line plot, Wilcoxon P < 0.0001. **(C)** Box plot comparison of Pre/Post PD-L1-positive CTC count and total CTC count. **(D)** PD-L1+/Total CTC ratio histogram. **(E)** Spearman correlation scatter plot (rho = 0.830, P < 0.001). **(F)** Waterfall plot of delta PD-L1+ CTC (Post minus Pre), colored by direction of change.

Immunofluorescence staining confirmed successful CTC enrichment and PD-L1 detection using the EpCAM antibody-modified microfluidic chip (**Figures 1A, B**). PD-L1-negative CTCs were identified by Hoechst+/PanCK+/CD45-/PD-L1- staining, while PD-L1-positive CTCs showed Hoechst+/PanCK+/CD45-/PD-L1+ staining, demonstrating clear discrimination with no signal overlap from leukocytes. Quantitative CTC distribution is shown in **Figure 2**. Pre-operative total CTCs were detected in 62/72 patients (86.1%), ranging from 0 to 12 per 4 mL (median = 6/4 mL; **Figure 2A**). Pre-operative PD-L1+ CTCs were detected in 41/72 patients (56.9%), ranging from 0 to 11 per 4 mL (median = 2/4 mL; **Figure 2B**). Pre-operative PD-L1- CTCs are shown in **Figure 2C**. Post-operative total CTCs were detected in 52/72 patients (72.2%), range 0-12/4 mL (median = 4/4 mL; **Figure 2D**). Post-operative PD-L1+ CTCs were detected in 33/72 patients (45.8%), range 0-9/4 mL (median = 0/4 mL; **Figure 2E**). Post-operative PD-L1- CTCs are shown in **Figure 2F**. Paired pre- and postoperative changes are presented in **Figure 3**. A significant postoperative decrease was observed in both PD-L1+ CTCs (Wilcoxon P < 0.0001; **Figure 3A**) and total CTCs (P < 0.0001; **Figure 3B**). Box plot comparison across all four groups is shown in **Figure 3C**. PD-L1 expression on CTCs was heterogeneous rather than an all-or-none phenomenon. Pre-operatively, all CTCs were PD-L1+ in 7 patients (9.7%), mixed PD-L1+/PD-L1- populations in 34 patients (47.2%), all CTCs PD-L1- in 21 patients (29.2%), and no CTCs detected in 10 patients (13.9%).

### 3.2 Correlation analysis between CTC PD-L1 expression and clinicopathological features of epithelial ovarian cancer

Correlations between CTC PD-L1 status and clinicopathological characteristics were analyzed using chi-square or Fisher’s exact tests (**Table 1**). No significant associations were observed between CTC PD-L1 status and age, histological subtype, FIGO stage, differentiation grade, lymph node metastasis, or CA125 levels (all P > 0.05).

**Table 1:** Correlation analysis between CTC PD-L1 expression and clinicopathological features in 72 patients with epithelial ovarian cancer before and after operation [n (%)].

Parameters	Cases, n	Preoperative CTC PD-L1 Detection		χ²	P value	Postoperative CTC PD-L1 Detection		χ²	P value
		PD-L1+ CTC, n (%)	PD-L1− CTC, n (%)			PD-L1+ CTC, n (%)	PD-L1− CTC, n (%)		
Age (years)				0.057	0.812			1.399	0.237
<60	36	20 (55.6%)	16 (44.4%)			14 (38.9%)	22 (61.1%)		
≥60	36	21 (58.3%)	15 (41.7%)			19 (52.8%)	17 (47.2%)		
Histopathological type				0.274	0.601			0.129	0.719
Serous adenocarcinoma	42	25 (59.5%)	17 (40.5%)			20 (47.6%)	22 (52.4%)		
Mucinous/Endometrioid/Clear cell	30	16 (53.3%)	14 (46.7%)			13 (43.3%)	17 (56.7%)		
Tumor differentiation				0.094	0.759			1.645	0.200
Poor differentiation	27	16 (59.3%)	11 (40.7%)			15 (55.6%)	12 (44.4%)		
Moderate/Well differentiation	45	25 (55.6%)	20 (44.4%)			18 (40.0%)	27 (60.0%)		
FIGO stage				0.030	0.863			0.076	0.782
Stage I/II	34	19 (55.9%)	15 (44.1%)			15 (44.1%)	19 (55.9%)		
Stage III/IV	38	22 (57.9%)	16 (42.1%)			18 (47.4%)	20 (52.6%)		
Lymph node metastasis				0.333	0.564			0.285	0.593
No	39	21 (53.8%)	18 (46.2%)			19 (48.7%)	20 (51.3%)		
Yes	33	20 (60.6%)	13 (39.4%)			14 (42.4%)	19 (57.6%)		
CA125				0.266	0.606			0.163	0.686
<35 U/mL	28	17 (60.7%)	11 (39.3%)			12 (42.9%)	16 (57.1%)		
≥35 U/mL	44	24 (54.5%)	20 (45.5%)			21 (47.7%)	23 (52.3%)		

CTCs, Circulating tumor cells; PD-L1, Programmed cell death-ligand 1; PD-L1+ CTCs, PD-L1-positive circulating tumor cells; PD-L1− CTCs, PD-L1-negative circulating tumor cells; FIGO stage, A clinical staging system developed by the International Federation of Gynecology and Obstetrics (FIGO), mainly used for ovarian cancer staging; CA125, Carbohydrate antigen 125.

### 3.3 CTC PD-L1 expression and prognostic analysis of epithelial ovarian cancer

**Figure 4:**
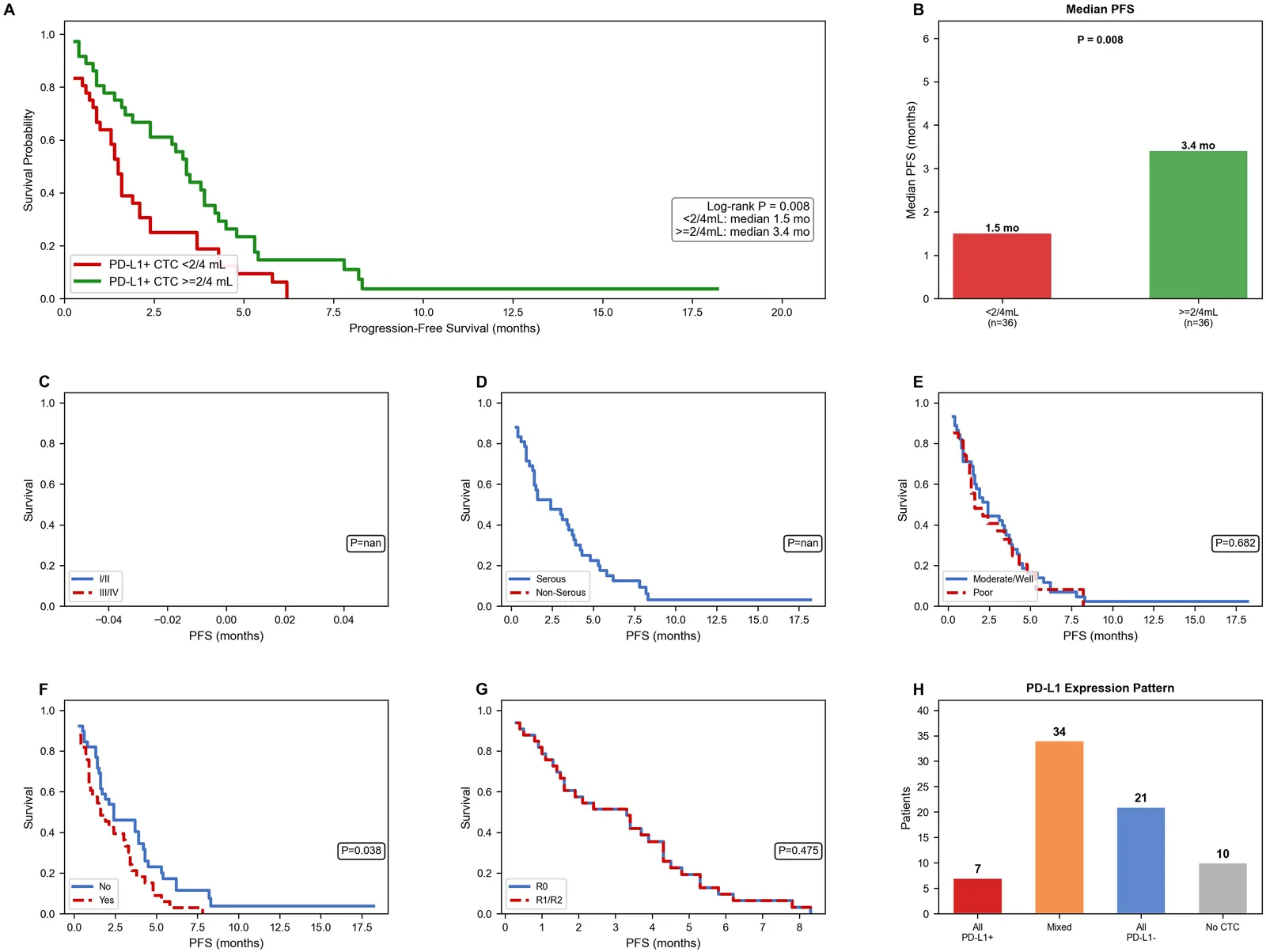
Kaplan-Meier estimates of progression-free survival stratified by preoperative PD-L1+ CTC count (n=72). **(A)** Main KM curve: PD-L1+ CTC <2/4 mL (red) vs ≥2/4 mL (green). Log-rank P = 0.008. **(B)** Median PFS comparison: <2/4 mL = 1.5 months, ≥2/4 mL = 3.3 months. **(C)** Subgroup KM: FIGO stage I/II vs III/IV. **(D)** Subgroup KM: Histology Serous vs Non-Serous. **(E)** Subgroup KM: Differentiation Moderate/Well vs Poor. **(F)** Subgroup KM: LN Metastasis No vs Yes. **(G)** Subgroup KM: Residual Disease R0 vs R1/R2. **(H)** PD-L1 expression pattern distribution (All PD-L1+ n=7, Mixed n=34, All PD-L1- n=21, No CTC n=10).

**Figure 5:**
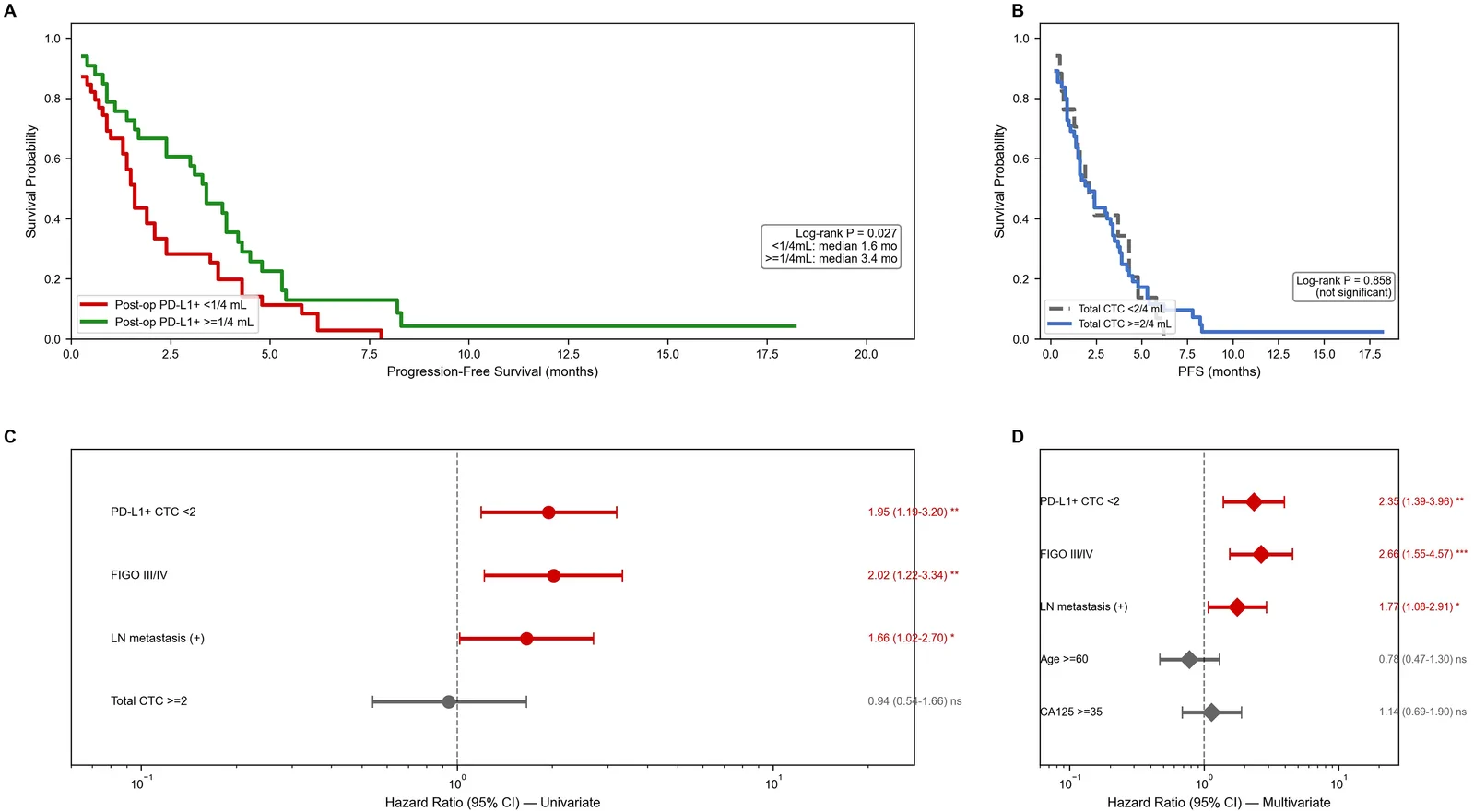
Post-operative analysis and Cox proportional hazards regression for progression-free survival (n=72). **(A)** Post-op PD-L1+ CTC KM curve (<1 vs ≥1/4 mL), Log-rank P = 0.027. **(B)** Total CTC KM curve (<2/4 mL vs ≥2/4 mL), Log-rank P = 0.858 (not significant). **(C)** Univariate Cox regression forest plot: PD-L1+ <2 HR = 1.95 (1.19-3.20)*; FIGO III/IV HR = 2.02 (1.22-3.34)**; LN+ HR = 1.66 (1.02-2.70)*; Total CTC≥2 HR = 0.94 (0.54-1.66)ns. **(D)** Multivariate Cox regression forest plot: PD-L1+ <2 aHR=2.35 (1.39-3.96)**; FIGO III/IV aHR=2.66 (1.55-4.57)***; LN+ aHR=1.77 (1.08-2.91)*; Age≥60 aHR=0.78ns; CA125≥35 aHR=1.14ns.

Kaplan-Meier survival analysis is presented in **Figures 4** and **5**. Patients with preoperative PD-L1+ CTC <2/4 mL had significantly shorter progression-free survival compared with ≥2/4 mL (median PFS: 1.5 vs 3.3 months; Log-rank P = 0.008; **Figure 4A**). Median PFS comparison is shown as a bar chart in **Figure 4B**. Subgroup KM analyses are presented in **Figures 4C–H**: FIGO stage I/II vs III/IV (**Figure 4C**), histology Serous vs Non-Serous (**Figure 4D**), differentiation Moderate/Well vs Poor (**Figure 4E**), lymph node metastasis No vs Yes (**Figure 4F**), residual disease R0 vs R1/R2 (**Figure 4G**), and PD-L1 expression pattern distribution (**Figure 4H**).

Post-operative PD-L1+ CTC KM curve is shown in **Figure 5A** (<1/4 mL vs ≥1/4 mL; Log-rank P = 0.027). Total CTC KM was not statistically significant (**Figure 5B**; Log-rank P = 0.858). Univariate Cox regression (**Figure 5C**) identified preoperative PD-L1+ CTC <2/4 mL (HR = 1.95, 95% CI: 1.19-3.20, P = 0.008) and FIGO stage III/IV (HR = 2.02, 95% CI: 1.22-3.34, P = 0.006) as significant predictors of PFS. Lymph node metastasis was also significant (HR = 1.66, 95% CI: 1.02-2.70, P = 0.040). Age, differentiation, CA125, and total CTC count were not significant.

Multivariate Cox regression (**Figure 5D**) confirmed preoperative PD-L1+ CTC <2/4 mL as an independent prognostic factor (adjusted HR = 2.35, 95% CI: 1.39-3.96, P = 0.001). FIGO stage III/IV was the strongest independent predictor (adjusted HR = 2.66, 95% CI: 1.55-4.57, P < 0.001). Lymph node metastasis remained significant (adjusted HR = 1.77, 95% CI: 1.08-2.91, P = 0.023). Age ≥60 years and CA125 ≥35 U/mL were not significant in the multivariate model.

Supplementary analyses are presented in **Supplementary Figures 1**–**4**. Total CTC count was not significantly associated with PFS in Kaplan-Meier analysis (**Supplementary Figure 1**; Log-rank P = 0.858). Complete Cox regression forest plots are shown in **Supplementary Figure 2** (univariate: **Supplementary Figure 2A**; multivariate: **Supplementary Figure 2B**). PD-L1 expression pattern analysis is presented in **Supplementary Figure 3**: preoperative pie chart (**Supplementary Figure 3A**), postoperative pie chart (**Supplementary Figure 3B**), PD-L1+/Total CTC ratio by group (**Supplementary Figure 3C**), scatter plot colored by expression pattern (**Supplementary Figure 3D**), pre- vs postoperative bar chart comparison (**Supplementary Figure 3E**), and delta PD-L1+ CTC changes colored by preoperative pattern (**Supplementary Figure 3F**). Subgroup Kaplan-Meier analyses are shown in **Supplementary Figure 4**: FIGO stage (**Supplementary Figure 4A**), histology (**Supplementary Figure 4B**), differentiation (**Supplementary Figure 4C**), and lymph node metastasis (**Supplementary Figure 4D**).

PD-L1+ CTC count correlated strongly with total CTC count (Spearman rho = 0.830, P < 0.001). Post-operative PD-L1+ CTC was not independently associated with PFS after multivariable adjustment.

### 3.4 Single-cell atlas reveals ovarian cancer cell heterogeneity and key cell types

**Figure 6:**
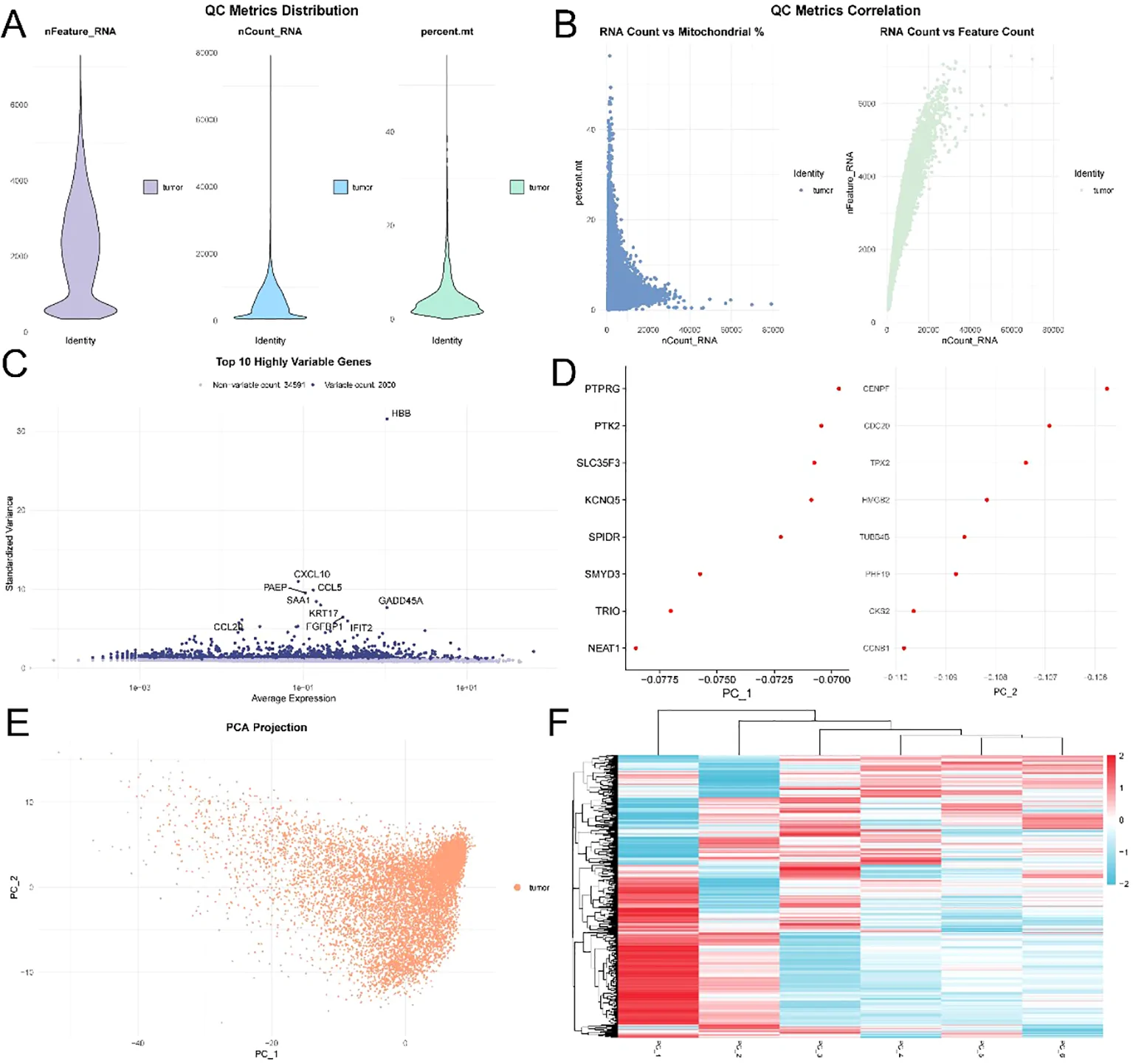
scRNA-seq data quality control, highly variable gene identification, and principal component analysis. **(A)** QC violin plots. **(B)** Feature scatter (nFeature_RNA vs nCount_RNA). **(C)** Highly variable genes (top 2000). **(D)** PC contribution elbow plot. **(E)** PCA projection (PC1 vs PC2). **(F)** Gene expression heatmap on principal components.

scRNA-seq quality control metrics are shown in **Figure 6** (17): QC violin plots (**Figure 6A**), feature scatter (nFeature_RNA vs nCount_RNA; **Figure 6B**), highly variable genes (top 2,000; **Figure 6C**), PC contribution elbow plot (**Figure 6D**), PCA projection (PC1 vs PC2; **Figure 6E**), and gene expression heatmap on principal components (**Figure 6F**).

**Figure 7:**
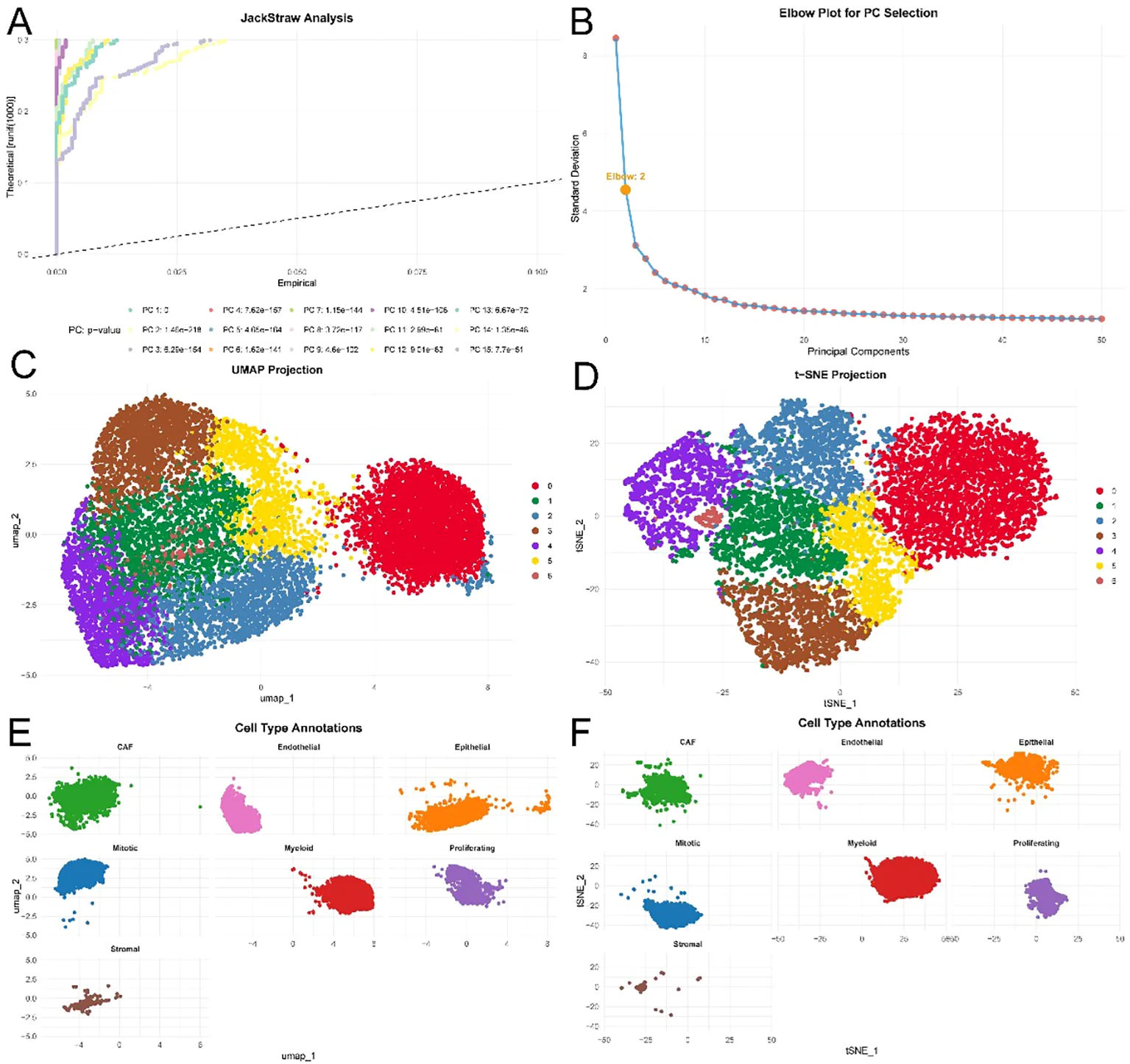
scRNA-seq data dimensionality reduction and cell type annotation. **(A)** JackStraw plot for PC significance. **(B)** Elbow plot for optimal PC selection. **(C)** UMAP projection by cluster. **(D)** t-SNE projection by cluster. **(E)** UMAP projection by cell type. **(F)** t-SNE projection by cell type. Seven cell types identified: CAF, Endothelial, Epithelial, Mitotic, Myeloid, Proliferating, Stromal.

Dimensionality reduction and cell type annotation are presented in **Figure 7**: JackStraw plot (**Figure 7A**), elbow plot for optimal PC selection (**Figure 7B**), UMAP by cluster (**Figure 7C**), t-SNE by cluster (**Figure 7D**), UMAP by cell type (**Figure 7E**), and t-SNE by cell type (**Figure 7F**). Seven distinct cell types were identified: cancer-associated fibroblasts (CAFs), endothelial cells, epithelial cells (presumed tumor cells), mitotic cells, myeloid cells, proliferating cells, and stromal cells.

### 3.5 Gene co-expression networks and role of telomere maintenance genes in ovarian cancer

**Figure 8:**
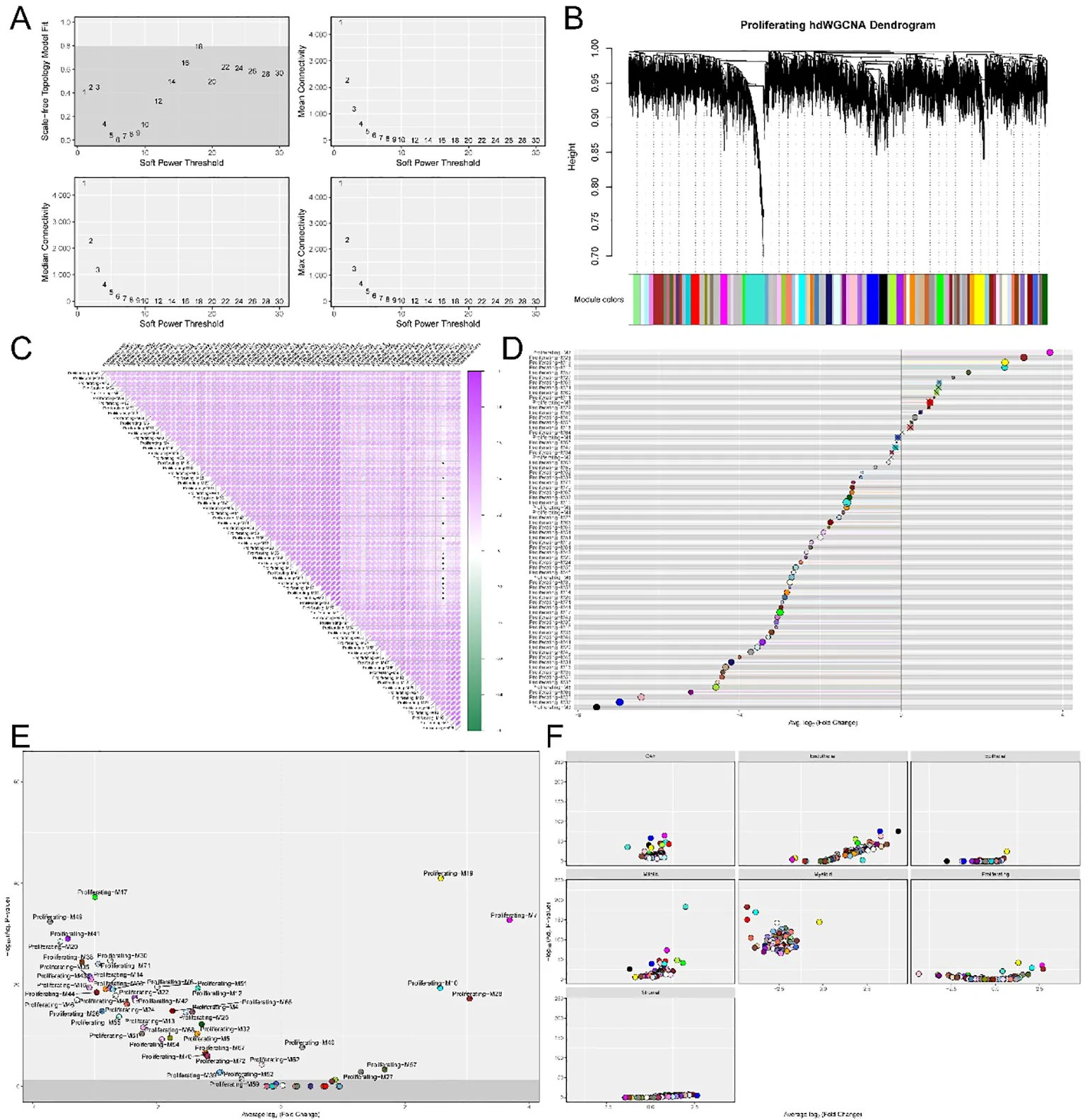
WGCNA soft power threshold selection, gene module identification, and differential expression analysis. **(A)** Soft threshold selection (scale-free topology fit). **(B)** Gene module dendrogram with color assignment. **(C)** Module-trait correlation heatmap. **(D)** Proliferation module average log2FC bar plot. **(E)** Volcano plot (log2FC vs -log10 P). **(F)** Cell-type-specific module volcano plots.

Gene co-expression network analysis is presented in **Figure 8**: soft power threshold selection (**Figure 8A**), gene module dendrogram (**Figure 8B**), module-trait correlation heatmap (**Figure 8C**), proliferation module log2FC bar plot (**Figure 8D**), volcano plot (**Figure 8E**), and cell-type-specific module volcano plots (**Figure 8F**).

**Figure 9:**
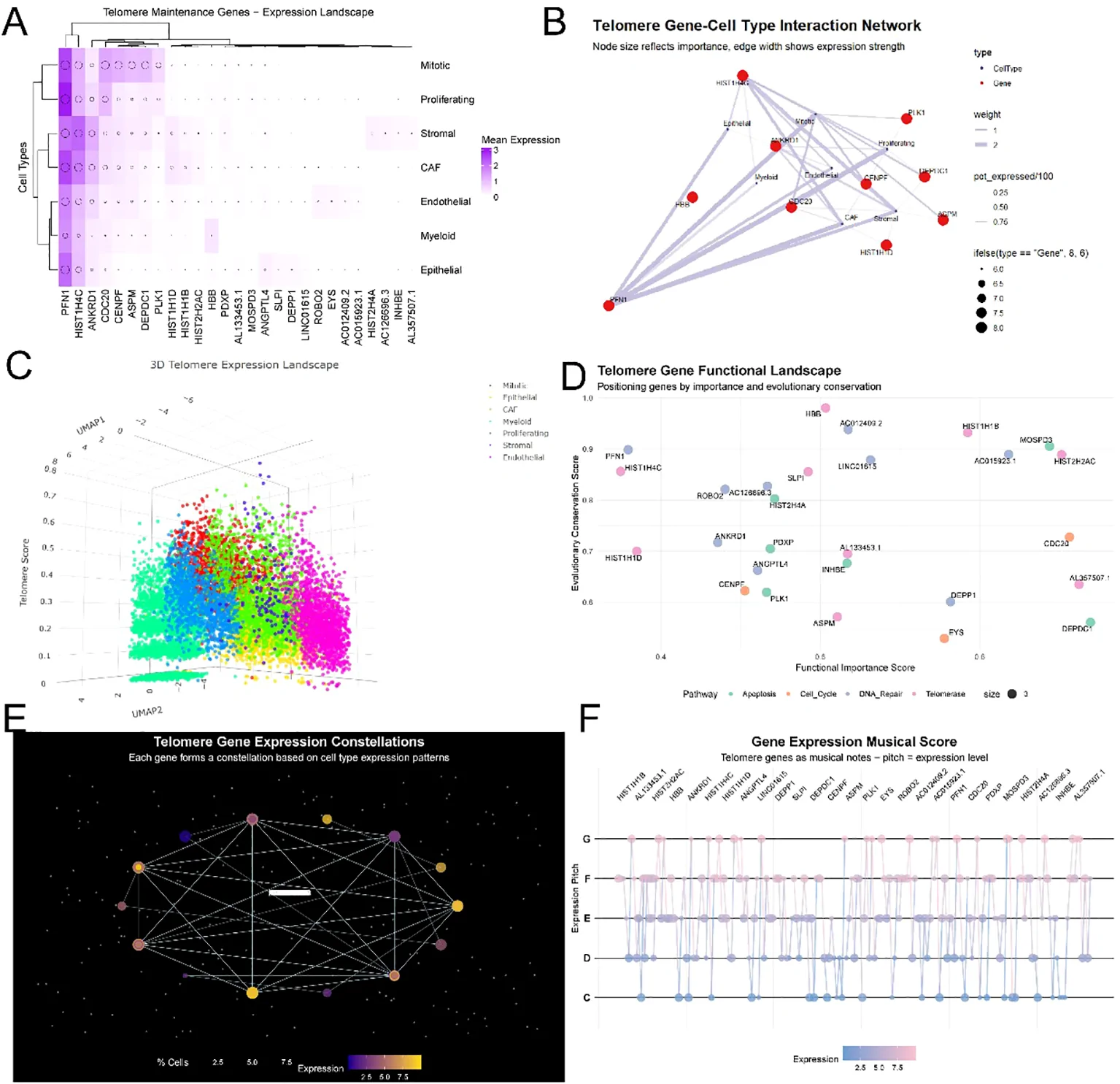
Telomere maintenance gene expression and functional analysis. **(A)** Expression heatmap of telomere genes across cell types. **(B)** Telomere gene-cell type interaction network. **(C)** 3D telomere expression landscape. **(D)** Telomere gene function landscape. **(E)** Gene expression constellation map. **(F)** Gene expression score map by cell type.

Telomere maintenance gene analysis is shown in **Figure 9**: expression heatmap across cell types (**Figure 9A**), telomere gene-cell type interaction network (**Figure 9B**), 3D expression landscape (**Figure 9C**), function landscape (**Figure 9D**), constellation map (**Figure 9E**), and expression score map (**Figure 9F**). Telomere maintenance genes exhibited distinct cell-type-specific patterns, with elevated activity in proliferating populations.

### 3.6 Pseudotime analysis reveals dynamic evolution of ovarian cancer cells and PD-L1-related gene expression

**Figure 10:**
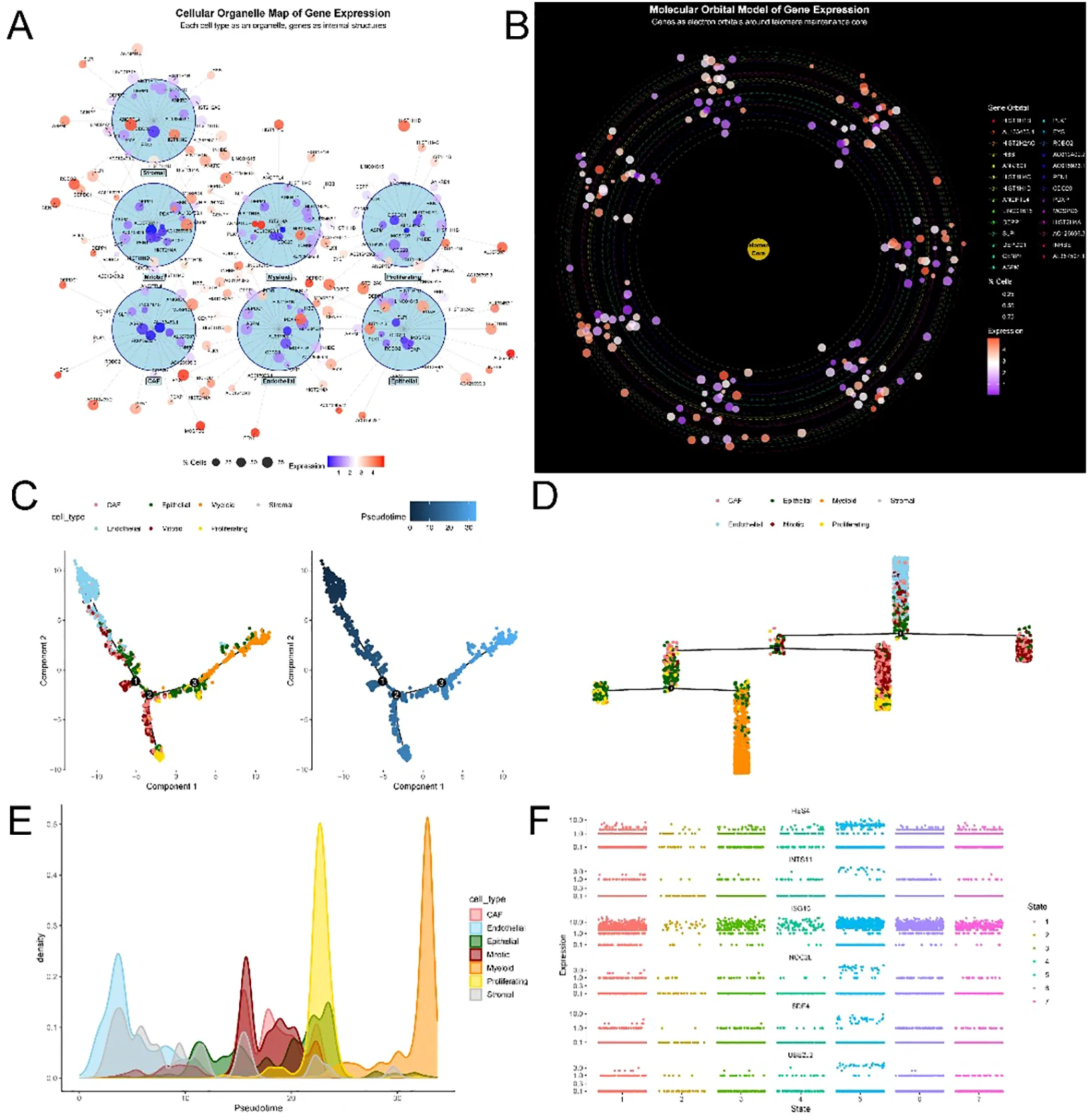
Cellular organelle map, molecular orbital model, and pseudotime trajectory analysis. **(A)** Cellular organelle map of gene expression. **(B)** Molecular orbital model of cell states. **(C)** Pseudotime trajectory (cells colored by pseudotime). **(D)** Cell type trajectories along pseudotime axis. **(E)** Cell type density distribution in pseudotime. **(F)** Gene expression dynamics along pseudotime (HES4, INTS11, ISG15, NOC2L, SDF4, UBE2J2).

Pseudotime trajectory analysis is presented in **Figure 10**: cellular organelle map (**Figure 10A**), molecular orbital model (**Figure 10B**), pseudotime trajectory with cells colored by progression (**Figure 10C**), cell type-specific trajectories (**Figure 10D**), density distribution in pseudotime (**Figure 10E**), and expression dynamics of specific genes (HES4, INTS11, ISG15, NOC2L, SDF4, UBE2J2) along pseudotime (**Figure 10F**), revealing dynamic patterns potentially related to proliferation, differentiation, or therapeutic response.

**Figure 11:**
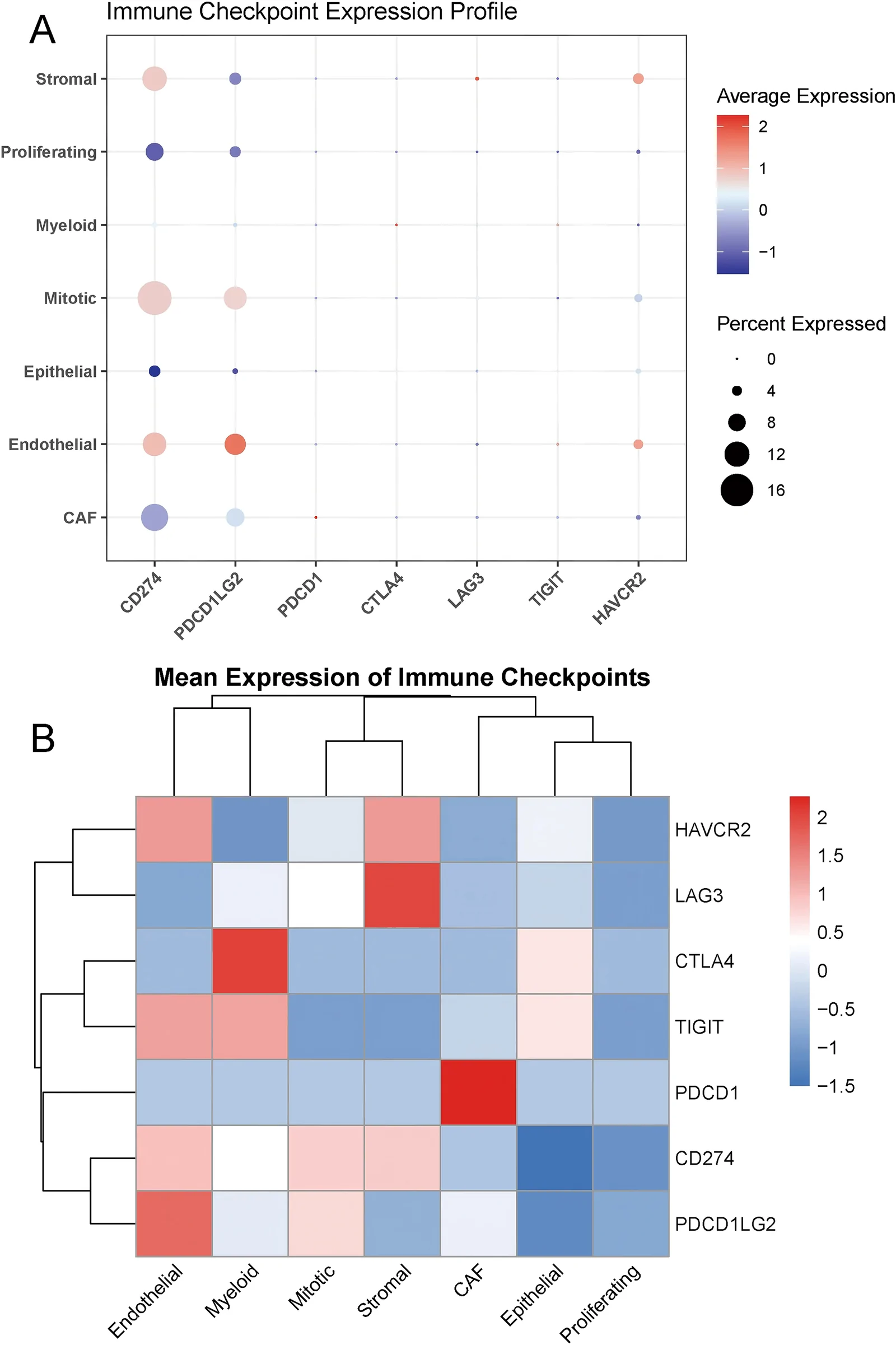
Single-cell expression analysis of PD-L1 and immune checkpoint genes in ovarian cancer. **(A)** Dot plot showing expression of key immune checkpoint genes (CD274/PD-L1, PDCD1, PDCD1LG2, CTLA4, LAG3, TIGIT, HAVCR2) across annotated ovarian cancer cell types: CAF, Endothelial, Epithelial, Mitotic, Myeloid, Proliferating, and Stromal cells. Dot size indicates the percentage of cells expressing the gene, and color intensity reflects mean expression level. **(B)** Heatmap of average expression of the same immune checkpoint genes across cell types. Red indicates higher expression, blue indicates lower expression. Hierarchical clustering highlights patterns of co-expression across cell populations.

Immune checkpoint gene expression is shown in **Figure 11**. Dot plot analysis (**Figure 11A**) of CD274/PD-L1, PDCD1, PDCD1LG2, CTLA4, LAG3, TIGIT, and HAVCR2 across the seven cell types revealed variable PD-L1 expression, with notable enrichment in stromal and myeloid compartments, whereas epithelial tumor cells showed lower average expression. Heatmap with hierarchical clustering (**Figure 11B**) revealed coordinated regulation of immune checkpoint pathways in stromal and myeloid populations, indicating cell-type-specific patterns of immune checkpoint gene expression.

### 3.7 IFN-γ induced upregulation of PD-L1 and immune-related genes across EOC cell lines

**Figure 12:**
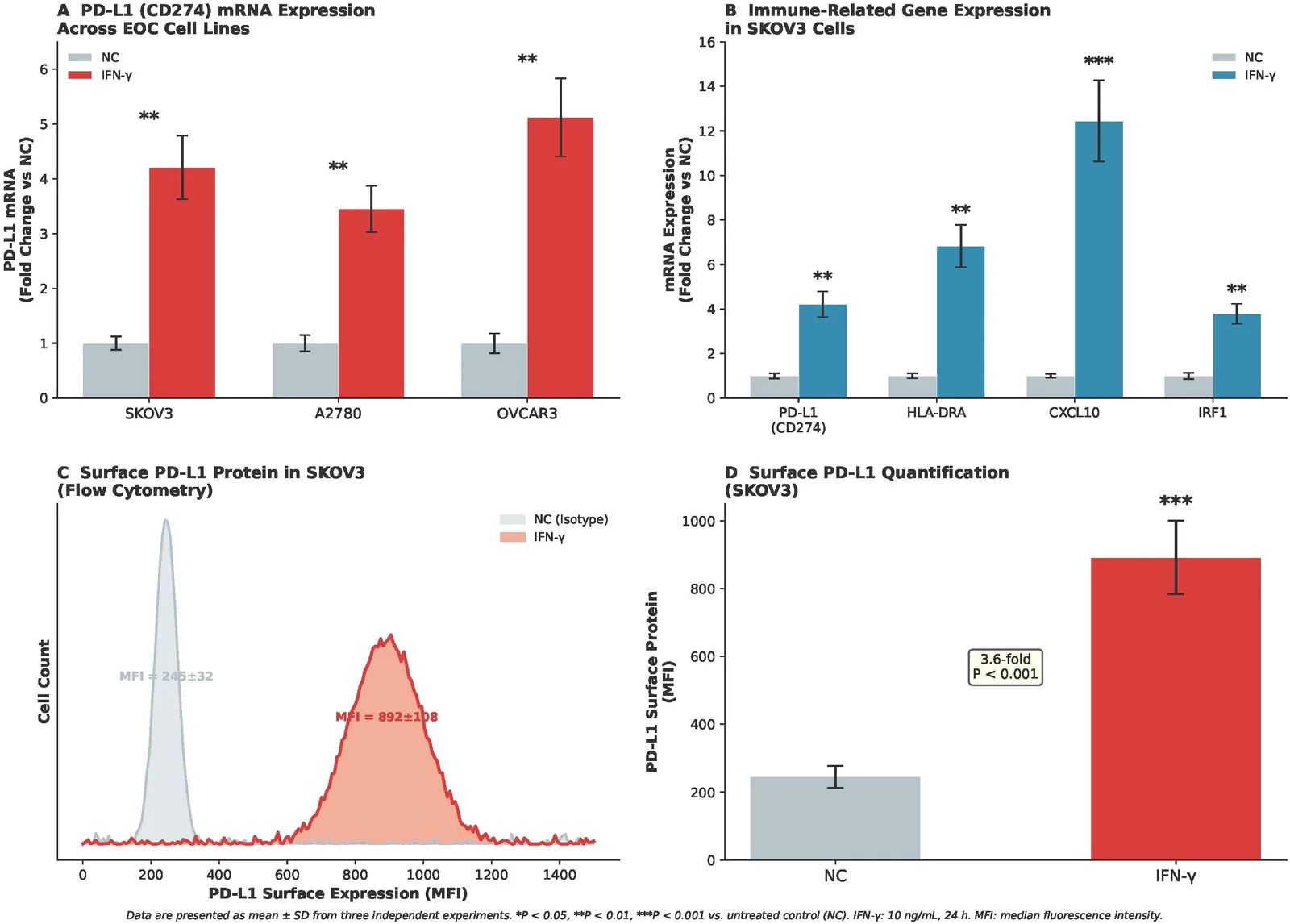
IFN-γ-induced PD-L1 expression across EOC cell lines at the mRNA and protein levels. **(A)** PD-L1 (CD274) mRNA expression in SKOV3, A2780, and OVCAR3 cells treated with IFN-γ (10 ng/mL, 24 h) vs. untreated control (NC). **(B)** mRNA expression of immune-related genes (PD-L1, HLA-DRA, CXCL10, IRF1) in SKOV3 cells. **(C)** Representative flow cytometry histograms of surface PD-L1 protein in SKOV3 cells (NC vs. IFN-γ). **(D)** Quantification of surface PD-L1 median fluorescence intensity (MFI). Data are presented as mean ± SD from three independent experiments. *P < 0.05, **P < 0.01, ***P < 0.001 vs. NC.

IFN-γ treatment (10 ng/mL, 24 h) significantly upregulated PD-L1 (CD274) mRNA across all three EOC cell lines tested: SKOV3 (4.2-fold, P < 0.01), A2780 (3.5-fold, P < 0.01), and OVCAR3 (5.1-fold, P < 0.01), indicating that IFN-γ-induced PD-L1 upregulation was observed in all three tested EOC cell lines (**Figure 12A**). In SKOV3 cells, IFN-γ also significantly upregulated HLA-DRA (6.8-fold, P < 0.01), CXCL10 (12.5-fold, P < 0.001), and the interferon regulatory factor IRF1 (3.8-fold, P < 0.01), a key transcription factor that mediates IFN-γ-induced PD-L1 transcription (18; **Figure 12B**). At the protein level, flow cytometry confirmed that IFN-γ significantly increased surface PD-L1 expression on SKOV3 cells (MFI: 245 vs. 892; 3.6-fold increase, P < 0.001; **Figures 12C, D**).

## 4 Discussion

Epithelial ovarian cancer (EOC) is currently the gynecological malignancy with the highest mortality rate, with a 5-year survival rate below 40.0% (1, 2, 19). More than 70.0% of EOC patients are at an advanced stage at diagnosis and may have peritoneal metastasis (20). Despite aggressive standard treatment with cytoreductive surgery combined with platinum-taxane-based chemotherapy (21, 22), the prognosis of EOC patients remains poor, with approximately 80.0% of EOC patients experiencing recurrence after treatment (20, 21). Insidious onset, tumor recurrence and metastasis, and poor sensitivity to radiotherapy and chemotherapy are the main reasons for poor prognosis in ovarian cancer, while early diagnosis is a key factor in improving the prognosis of epithelial ovarian cancer. A small number of domestic and international studies have confirmed that circulating tumor cells (CTCs) may have auxiliary diagnostic value in prognostic evaluation of epithelial ovarian cancer.

During the process of CTCs entering the blood circulation, epithelial-mesenchymal transition (EMT) occurs, during which tumor cells lose epithelial markers and acquire mesenchymal characteristics (23). This phenotypic plasticity (23) limits the sensitivity of conventional EpCAM-only CTC capture methods (24). Abreu et al. reported that EpCAM-based immunomagnetic separation achieved a CTC detection rate of only 21% in advanced high-grade serous ovarian cancer (25). Molecular characterization of CTCs has been explored through gene expression panels (26). Prior studies have compared EpCAM-based enrichment strategies in ovarian cancer (27). In the present study, the EpCAM antibody-based HB-Chip achieved a preoperative CTC detection rate of 86.1% (62/72). However, we acknowledge that the current study did not include formal analytical validation (e.g., spike-in recovery experiments). Published validation studies for the HB-Chip platform have reported capture efficiencies of 85-95% for cancer cell lines (15, 16), but direct comparative studies in ovarian cancer remain to be performed. Future studies should include head-to-head comparisons, recovery rate determination, and inter-operator reproducibility assessment.

CTC PD-L1 expression detection can assist in prognostic evaluation of tumor surgical treatment. Manjunath et al. found that CTC PD-L1 expression is a survival predictor in NSCLC patients (28). Liu et al. showed that PD-L1-positive CTCs may serve as clinically relevant prognostic markers in gastric cancer patients (29). To our knowledge, studies evaluating the prognostic significance of CTC PD-L1 expression specifically in EOC remain limited. In our study, preoperative PD-L1+ CTC count was an independent prognostic factor for PFS in multivariate Cox regression (adjusted HR = 2.35, P = 0.001). Notably, patients with lower PD-L1+ CTC counts had worse prognosis. While counterintuitive at first glance, this finding is biologically plausible: tumors with higher PD-L1 expression may elicit stronger host anti-tumor immune responses, which could result in better clinical outcomes (30). Importantly, total CTC count was not significantly associated with PFS, suggesting that PD-L1 expression status on CTCs, rather than CTC quantity alone, carries the prognostic information (31). These tissue-based single-cell findings provide complementary biological context for the observed association between PD-L1-positive CTCs and PFS. However, because the scRNA-seq dataset was derived from primary tumor tissue rather than matched CTC samples, it does not directly establish the cellular origin or prognostic function of PD-L1-positive CTCs.

In summary, this study confirms that EpCAM antibody-based microfluidic chip technology can achieve CTC enrichment and PD-L1 expression detection in epithelial ovarian cancer. Pre-operative PD-L1+ CTC count is an independent prognostic factor for progression-free survival after adjusting for established clinical variables. Re-analysis of publicly available single-cell RNA sequencing data further revealed ovarian cancer cell heterogeneity (32). The *in vitro* validation across multiple EOC cell lines provides supportive cellular-level evidence for dynamic, immune-mediated regulation of PD-L1 at both the mRNA and surface protein levels. The growing clinical importance of immune checkpoint inhibitors (ICIs) warrants discussion of the translational relevance of these findings (33). Several clinical trials have identified subsets of ovarian cancer patients who derive benefit from ICIs, including the JAVELIN Ovarian 200 trial of avelumab in platinum-resistant disease (34), the KEYNOTE-100 trial of pembrolizumab with higher response rates in PD-L1-positive tumors (35), and the NRG GY003 trial of nivolumab plus ipilimumab (36). In this context, CTC PD-L1 detection may offer a dynamic, non-invasive approach for immunotherapy patient selection and monitoring. Several limitations should be acknowledged: (1) single-center design (n=72) limits generalizability; (2) formal analytical validation of the CTC platform (spike-in recovery, inter-operator reproducibility) was not performed; (3) immunofluorescence image quality in **Figure 1** is limited by the available microscopy and image acquisition settings, which may affect independent assessment of staining specificity—future studies should employ higher-resolution imaging with standardized acquisition parameters; (4) the scRNA-seq analysis was performed on a publicly available dataset (GEO: GSM8072347) from primary ovarian cancer tissue, not on CTCs from the clinical cohort; (5) PD-L1+ CTC cutoffs were data-driven and require external validation; (6) overall survival data are not yet mature; (7) *in vitro* experiments, while expanded to three EOC cell lines with both mRNA and surface protein-level validation, used established cell lines rather than patient-derived CTCs and did not include pathway-specific inhibition. Despite these limitations, the integration of CTC liquid biopsy, single-cell transcriptomic re-analysis, and *in vitro* validation represents a multi-dimensional approach (37) supporting the prognostic value of CTC PD-L1 detection in EOC. The EpCAM-based CTC enrichment and PD-L1 detection approach warrants further investigation in larger, multi-center prospective studies to validate its clinical utility.

## Data Availability

The datasets presented in this study can be found in online repositories. The names of the repository/repositories and accession number(s) can be found in the article/**Supplementary Material**.

## Supplementary material

Supplementary Figure 1 Kaplan-Meier estimates of progression-free survival stratified by preoperative total CTC count (n=72). Total CTC <2/4 mL vs ≥2/4 mL. Log-rank P = 0.858 (not significant).

Supplementary Figure 2 Forest plots of Cox proportional hazards regression for progression-free survival. **(A)** Univariate analysis. **(B)** Multivariate analysis (adjusted for age, FIGO stage, lymph node metastasis, and CA125). Hazard ratios (diamonds) with 95% confidence intervals (horizontal lines). Red: P < 0.05; Gray: P ≥ 0.05.

Supplementary Figure 3 PD-L1 expression pattern analysis of CTCs (n=72). **(A)** Pre-operative pie chart (All PD-L1+ n=7, Mixed n=34, All PD-L1- n=21, No CTC n=10). **(B)** Post-operative pie chart. **(C)** PD-L1+/Total CTC ratio box plot by PD-L1 group. **(D)** Scatter plot of Total CTC vs PD-L1+ CTC colored by expression pattern. **(E)** Pre vs Post bar chart comparison of pattern counts. **(F)** Delta PD-L1+ CTC scatter (Post minus Pre) colored by preoperative pattern.

Supplementary Figure 4 Subgroup Kaplan-Meier analyses of progression-free survival. **(A)** FIGO stage I/II vs III/IV. **(B)** Histology Serous vs Non-Serous. **(C)** Differentiation Moderate/Well vs Poor. **(D)** Lymph node metastasis No vs Yes.
