# Supplementary material for: Single-cell analysis and EpCAM-based microfluidic chip detection of PD-L1 on circulating tumor cells for prognostic prediction in epithelial ovarian cancer
Source: Front Oncol. 2026 Aug 10;16:1824350. doi: 10.3389/fonc.2026.1824350 (PMC13500509; doi:10.3389/fonc.2026.1824350)

Supplementary Figure 1

Kaplan-Meier estimates of progression-free survival stratified by preoperative total CTC count (n=72). Total CTC <2/4 mL vs >=2/4 mL. Log-rank P = 0.858 (not significant).

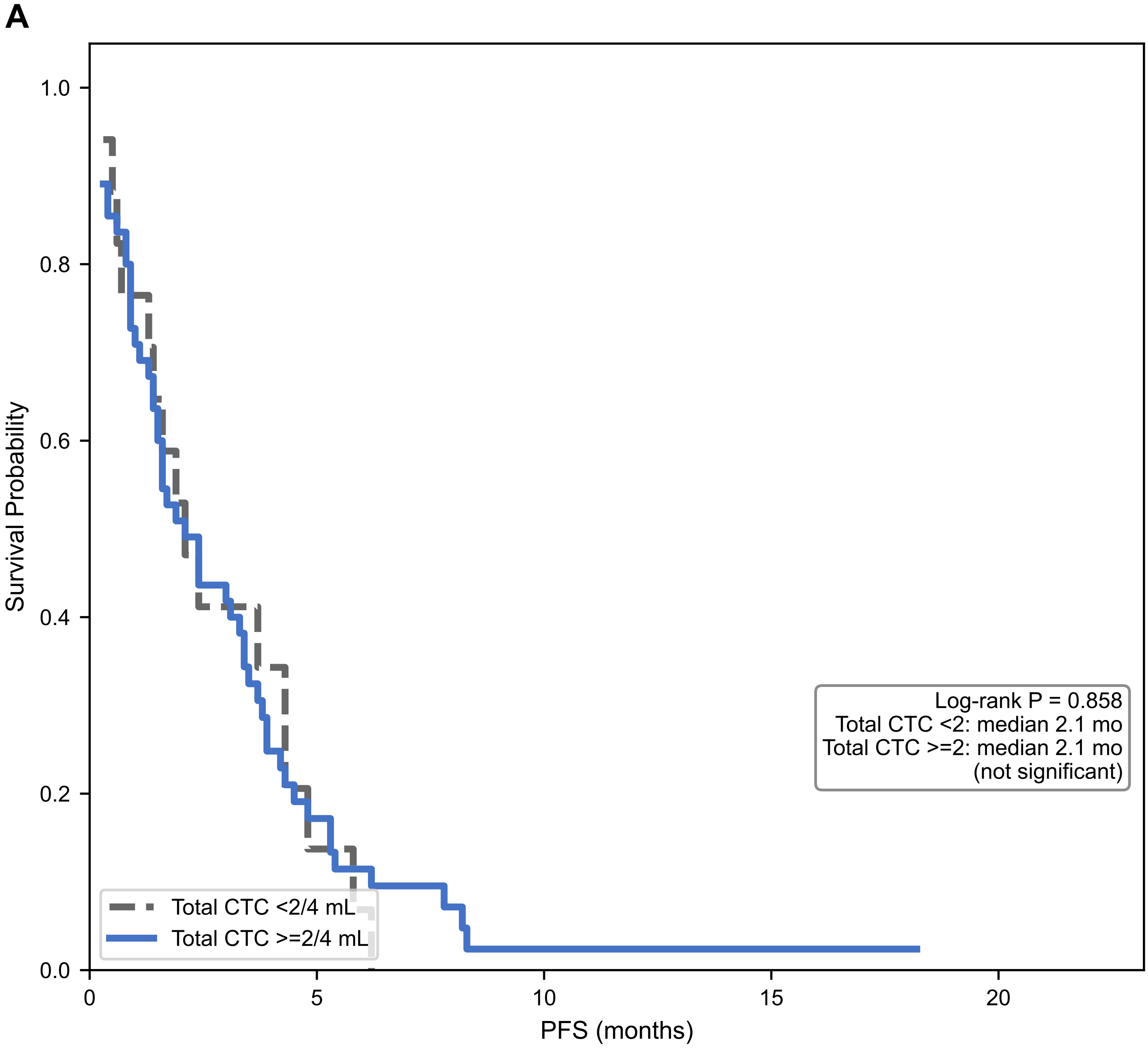

Supplementary Figure 2

Forest plots of Cox proportional hazards regression for progression-free survival. (A) Univariate analysis. (B) Multivariate analysis (adjusted for age, FIGO stage, lymph node metastasis, and CA125). Hazard ratios (diamonds) with 95% confidence intervals (horizontal lines). Red: P < 0.05; Gray: P >= 0.05.

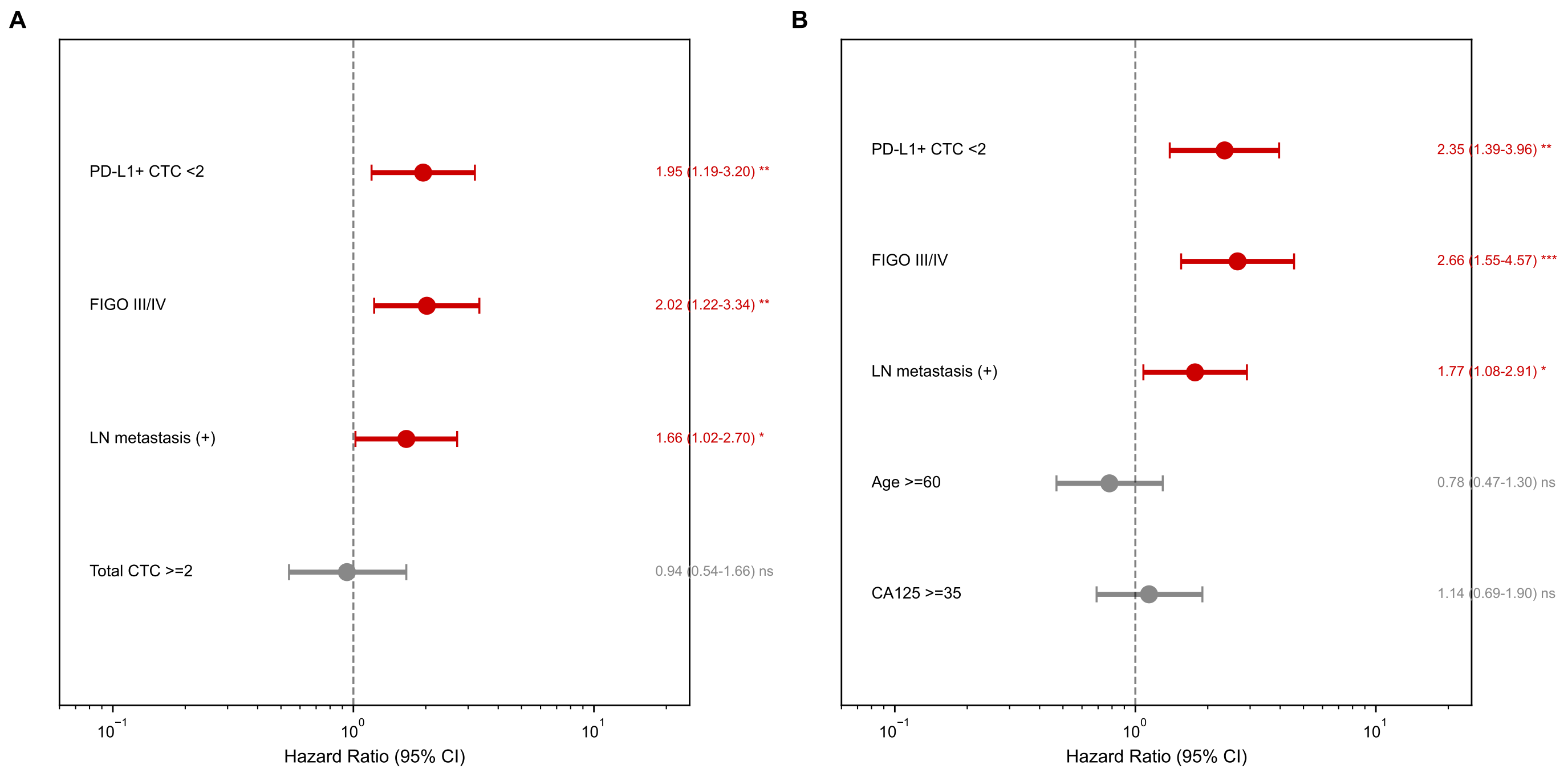

Supplementary Figure 3

PD-L1 expression pattern analysis of CTCs (n=72). (A) Pre-operative pie chart (All PD-L1+ n=7, Mixed n=34, All PD-L1- n=21, No CTC n=10). (B) Post-operative pie chart. (C) PD-L1+/Total CTC ratio box plot by PD-L1 group. (D) Scatter plot of Total CTC vs PD-L1+ CTC colored by expression pattern. (E) Pre vs Post bar chart comparison of pattern counts. (F) Delta PD-L1+ CTC scatter (Post minus Pre) colored by preoperative pattern.

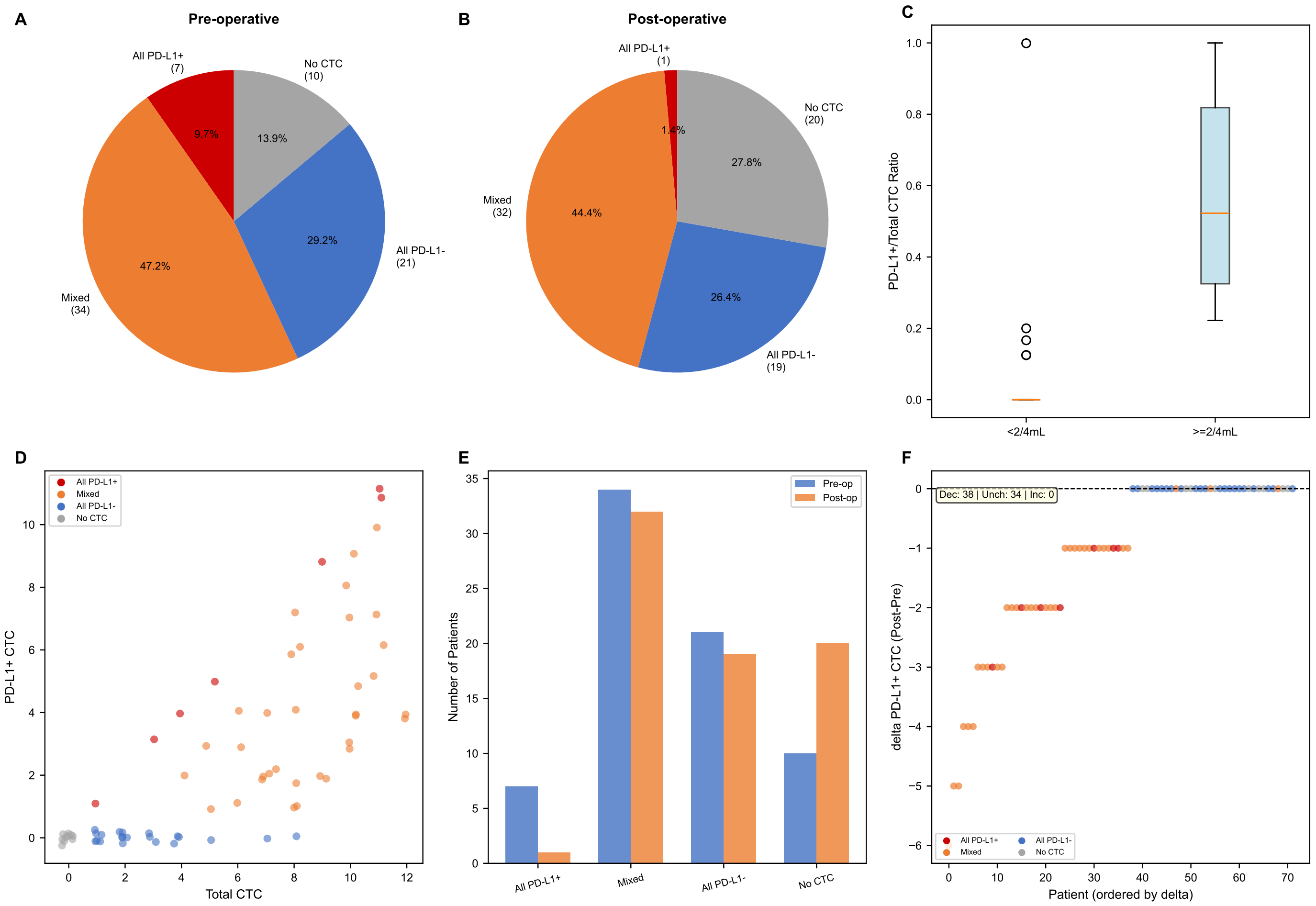

Supplementary Figure 4

Subgroup Kaplan-Meier analyses of progression-free survival. (A) FIGO stage I/II vs III/IV. (B) Histology Serous vs Non-Serous. (C) Differentiation Moderate/Well vs Poor. (D) Lymph node metastasis No vs Yes.

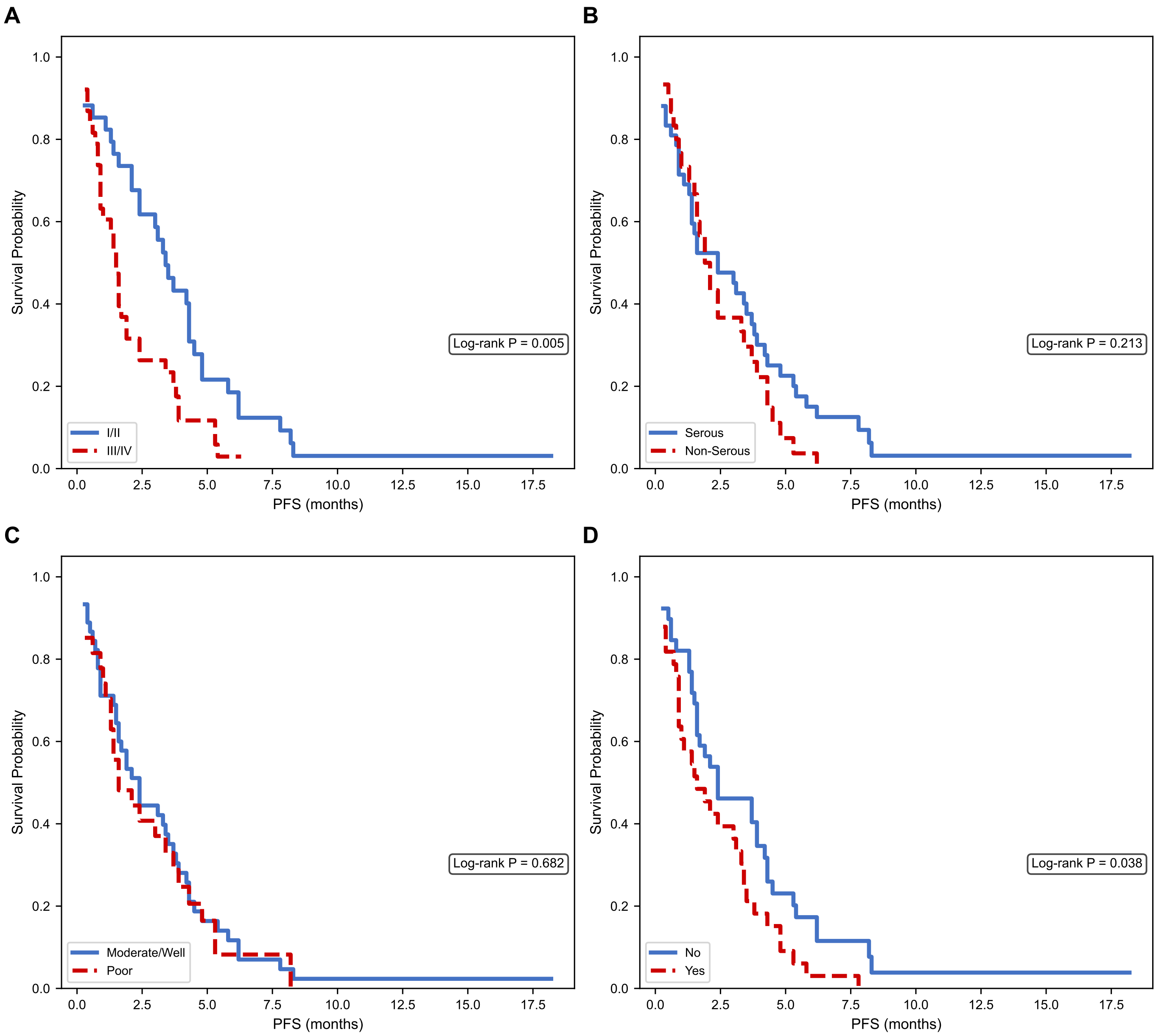

Supplement: Supplementary file 1 [file SupplementaryFile1.pdf]
